# Social media behavior is associated with vaccine hesitancy

**DOI:** 10.1093/pnasnexus/pgac207

**Published:** 2022-09-30

**Authors:** Steve Rathje, James K He, Jon Roozenbeek, Jay J Van Bavel, Sander van der Linden

**Affiliations:** Department of Psychology & Center for Neural Science, New York University, New York, NY 10003,USA; Department of Psychology, University of Cambridge, Cambridge CB2 3RQ, UK; Department of Psychology, University of Cambridge, Cambridge CB2 3RQ, UK; Department of Psychology & Center for Neural Science, New York University, New York, NY 10003,USA; Department of Psychology & Center for Neural Science, New York University, New York, NY 10003,USA

**Keywords:** vaccine hesitancy, misinformation, social media, social networks, echo chamber

## Abstract

Understanding how vaccine hesitancy relates to online behavior is crucial for addressing current and future disease outbreaks. We combined survey data measuring attitudes toward the COVID-19 vaccine with Twitter data in two studies (*N*_1_ = 464 Twitter users, *N*_2_ = 1,600 Twitter users) with preregistered hypotheses to examine how real-world social media behavior is associated with vaccine hesitancy in the United States (US) and the United Kingdom (UK). In Study 1, we found that following the accounts of US Republican politicians or hyper-partisan/low-quality news sites were associated with lower confidence in the COVID-19 vaccine—even when controlling for key demographics such as self-reported political ideology and education. US right-wing influencers (e.g. Candace Owens, Tucker Carlson) had followers with the lowest confidence in the vaccine. Network analysis revealed that participants who were low and high in vaccine confidence separated into two distinct communities (or “echo chambers”), and centrality in the more right-wing community was associated with vaccine hesitancy in the US, but not in the UK. In Study 2, we found that one's likelihood of not getting the vaccine was associated with retweeting and favoriting low-quality news websites on Twitter. Altogether, we show that vaccine hesitancy is associated with following, sharing, and interacting with low-quality information online, as well as centrality within a conservative-leaning online community in the US. These results illustrate the potential challenges of encouraging vaccine uptake in a polarized social media environment.

Significance Statement There has been extensive speculation that a social media “infodemic” may be contributing to vaccine hesitancy. Yet, little work has examined the crucial link between social media behavior and public health attitudes. We tested how self-reported attitudes about the COVID-19 vaccine relate to real-world social media behavior. We find that following, sharing, or interacting with low-quality news sources—as well as being a part of a conservative “echo chamber” in the US—is associated with vaccine hesitancy. These results should help researchers and policymakers understand online communities associated with vaccine hesitancy and inform solutions for encouraging vaccine uptake.

## Introduction

Mitigating the COVID-19 pandemic and preventing future disease outbreaks requires understanding and overcoming vaccine hesitancy (1, 2). Many have expressed concern that a misinformation “infodemic” on social media platforms such as Facebook and Twitter may contribute to vaccine hesitancy (3–6). Indeed, the US Surgeon General has called vaccine misinformation on social media an “urgent threat to public health” (7) and US President Joe Biden has insinuated that platforms such as Facebook are “killing people” with vaccine misinformation (8). In the current work, we examine the potential link between social media behavior and vaccine hesitancy during the COVID-19 pandemic.

Past research has linked misinformation exposure to vaccine hesitancy. For instance, endorsement of COVID-19 misinformation is associated with reduced intentions to get vaccinated for COVID-19 (9, 10, 11), and exposure to COVID-19 misinformation can lead to a causal reduction in intentions to receive the vaccine (12). This is potentially deadly, as anti-vaccination viewpoints have been growing steadily on social media platforms such as Facebook (4). Indeed, one survey found that people who get their news primarily from Facebook were more vaccine-hesitant than a number of other groups—including those who get their news primarily from Fox News (13). Altogether, these results indicate that exposure to misinformation on social media may have detrimental effects for vaccine uptake.

Other research has found that attitudes about the vaccine and COVID-19 have been strongly politicized, particularly in the United States (US). US conservatives report higher levels of vaccine hesitancy (11, 14), and the right-leaning media in the US have disproportionately shared misinformation about COVID-19 (15). An analysis of mobility data for 15 million Americans found that voting for Trump and watching Fox News were two of the biggest predictors of not complying with social distancing regulations during the pandemic (16). Additionally, exposure to cues from party elites (e.g. Trump or Biden promoting the vaccine) can causally influence vaccination intentions (17). However, vaccination attitudes and COVID-19 prevention behaviors have not been strongly related to conservatism in most other countries (11, 18, 19–21), indicating messages from political elites (22), rather than conservative ideology on its own, may have played a unique role in politicizing attitudes about the vaccine.

Social media also tends to reflect “echo chambers” in which people are selectively exposed to like-minded opinions (3, 23) and form social ties with likeminded others (24). Though it should be noted that homophily—or seeking out like-minded others—is present on domains outside of social media (25); for instance, partisans also sort into neighborhoods with co-partisans (26, 27). In fact, there is debate about how strong “echo chambers” are on social media (28). Just as people might exist in political “echo chambers” online and offline, it is possible that people with vaccine hesitant attitudes also congregate in “echo chambers”, hearing views only from people with similar beliefs. If this is true, it could undermine public health efforts that try to encourage vaccine uptake, since people who are part of antivaccine “echo chambers” may not be exposed to accurate information about the vaccine or efforts to correct vaccine misinformation, for example via fact-checks (29).

While it is important to understand the role of social media in shaping vaccine beliefs, most prior research has examined the predictors of vaccine hesitancy using either survey data or social media data on their own. To better understand how online behavior is related to vaccine attitudes beyond self-reported variables, we combined survey data with social media data. This allows us to have a more precise look at how real-world social media behavior is associated with beliefs about vaccination.

## Overview

To understand how social media behavior is related to vaccine hesitancy, we collected two samples of survey data about vaccine attitudes linked to Twitter data. Based on the prior literature, we tested four preregistered hypotheses (see https://aspredicted.org/8hp2q.pdf for the preregistration):

H1: The number of conservative politicians one follows will be negatively associated with vaccine confidence.
H2: The number of hyper-partisan/low-quality news sites one follows will be negatively associated with vaccine confidence.
H3: People with high and low levels of vaccine confidence will cluster into online “echo chambers.”
H4: People with lower vaccine confidence will share more hyper-partisan and low-quality news articles.

To test these hypotheses, we conducted two studies where we collected survey data linked to Twitter data. In Study 1 (*n* = 464 Twitter handles), we collected a roughly politically-balanced sample of liberals and conservatives from the United Kingdom (UK) and the US along with a sample of participants who specifically reported being vaccine hesitant. In this sample, we used regression models to examine whether the number of conservative politicians and hyper-partisan websites one follows predicted vaccine hesitancy. Standardized beta coefficients are reported for all regression models for ease of interpretation, and regression models were all run with and without demographic control variables. Then, we conducted network analysis to examine whether vaccine hesitant and vaccine-confident participants clustered into “echo chambers” in the US and the UK. In Study 2, we recruited a convenience sample (*n* = 1600) of participants via a web app called “Have I Shared Fake News.” Using this larger sample, we tested whether vaccine hesitancy predicted sharing and engaging with lower-quality information on social media in regression models.

## Study 1

For Study 1, we collected a total sample of 1,246 participants via the survey platform Prolific Academic from 2021 May 11, to 2021 June 29. To recruit a large enough sample of vaccine-hesitant participants, as well as politically diverse participants, we used the survey platform's prescreening options to oversample vaccine-hesitant and vaccine-neutral participants. We also aimed for a roughly equal number of participants from the US and the UK. See the “Materials and methods” Section for details about study recruitment.

Participants completed a two-item measure of COVID-19 vaccine confidence asking, on a scale of 1 to 7 (from 1 = “strongly disagree” to 7 = “strongly agree”) whether “the currently available COVID-19 vaccines are. . .” (1) effective and (2) safe (α = 0.97, *M* = 5.35, SD = 3.29). Participants completed a one-item measure asking if they intend to receive the COVID-19 vaccine, and a one-item measure indicating their political ideology on a scale of 1 to 7 (from 1 = “very liberal/very left-wing” to 7 = “very conservative/very right-wing”) (*M* = 3.97, SD = 1.95). Participants also completed a measure indicating whether they had or intended to receive the COVID-19 vaccine (896 yes, 349 no, 35 missing) and a number of demographic questions. See Supplementary Appendix Materials Section S1 for full question wording.

A total of 587 participants voluntarily provided their Twitter handles (30), and we were able to scrape 464 follower networks for analysis (175 M, 207 F, 6 transgender/non-binary/other, 73 Missing; *M*_age_ = 37.7; SD_age_ = 12.5). A total of 157 handles were from participants who reported being from the US, and 223 handles were from participants who reported being from the UK (the other 81 participants provided no answer or reported other countries). A total of 118 participants reported that they did not intend to get the COVID-19 vaccine, while 342 reported that they intended to get the vaccine. See Supplementary Appendix Table S1 for details about demographics across all samples.

### Following behavior and vaccine confidence

We first tested whether following conservative politicians was negatively associated with vaccine confidence (H1). We found that the number of US Republican politicians an individual followed on Twitter [from a list of the Twitter handles of 331 US Republicans adapted from ref. (31)] negatively predicted confidence in the COVID-19 vaccine, *β* = −0.12, 95% CI = [−0.21, −0.03], *P* = 0.011. Interestingly, this pattern still held in a multiple regression adjusting for self-reported political ideology, age, gender, education (e.g. having a Bachelor's degree), number of Twitter followers, and number of accounts followed, *β* = −0.18, 95% CI = [−0.30, −0.05], *P* = 0.006 (see Supplementary Appendix Table S2 for full models). However, the number of accounts followed by individuals from a list of UK Conservative Party politicians did not predict vaccine confidence, *β* = 0.06, 95% CI = [−0.04, 0.15], *P* = 0.230 (with control variables), *β* = 0.03, 95% CI = [−0.10, 0.16], *P* = 0.663. In sum, following US Republican politicians (but not UK conservative politicians) predicts vaccine hesitancy—even after adjusting for several covariates. These results are in support of H1 in the US, but not the UK. We did not specifically preregister predictions regarding differences in the US and the UK, but this observation is consistent with other research on differences in polarization about vaccination and COVID-19 in the US and the UK (11, 19, 20).

We then tested whether following Twitter accounts associated with “hyper-partisan” websites negatively predicted vaccine confidence (H2). “Hyper-partisan” websites refer to websites that are rated as low-quality by independent fact-checkers (32) and often share highly partisan (though not always false) content (e.g. “Breitbart”). These websites tend to be much more common than “fake news” websites that share completely fabricated content (33). The number of hyper-partisan Twitter accounts a participant followed [out of a list of 32 hyper-partisan Twitter handles adapted from ref. (32)] also negatively predicted vaccine confidence, *β* = −0.15, 95% CI = [−0.24, −0.06], *P* = 0.002. This result once again held even after adjusting for self-reported ideology, age, and gender, *β* = −0.20, 95% CI = [−0.32, −0.08], *P* = 0.002.

As a robustness check, we ran the same analysis using a larger list of 516 Twitter handles of news sites that were rated as untrustworthy by *NewsGuard* (34, 35), which has a team of journalists rate the quality of news websites on a scale of 1 to 100 (low-quality websites have a rating below 60). This broader list of news sites did not necessarily contain only hyper-partisan news, but also celebrity gossip sites (e.g. “TMZ”), alternative health sites, and more, as well as non-English and non-US-based sites. Once again, the number of Twitter handles of untrustworthy news sites one followed negatively predicted self-reported vaccine confidence, *β* = −0.19, 95% CI = [−0.28, −0.10], *P* < 0.001, including when adjusting for covariates, *β* = −0.19, 95% CI = [−0.31, −0.06], *P* = 0.003. Thus, following low-quality or hyper-partisan news sources predicts vaccine hesitancy even when accounting for political ideology and education, in support of H2. See Supplementary Appendix Table S3 and 4 for additional robustness checks.

### Network analysis

**Fig. 1.**
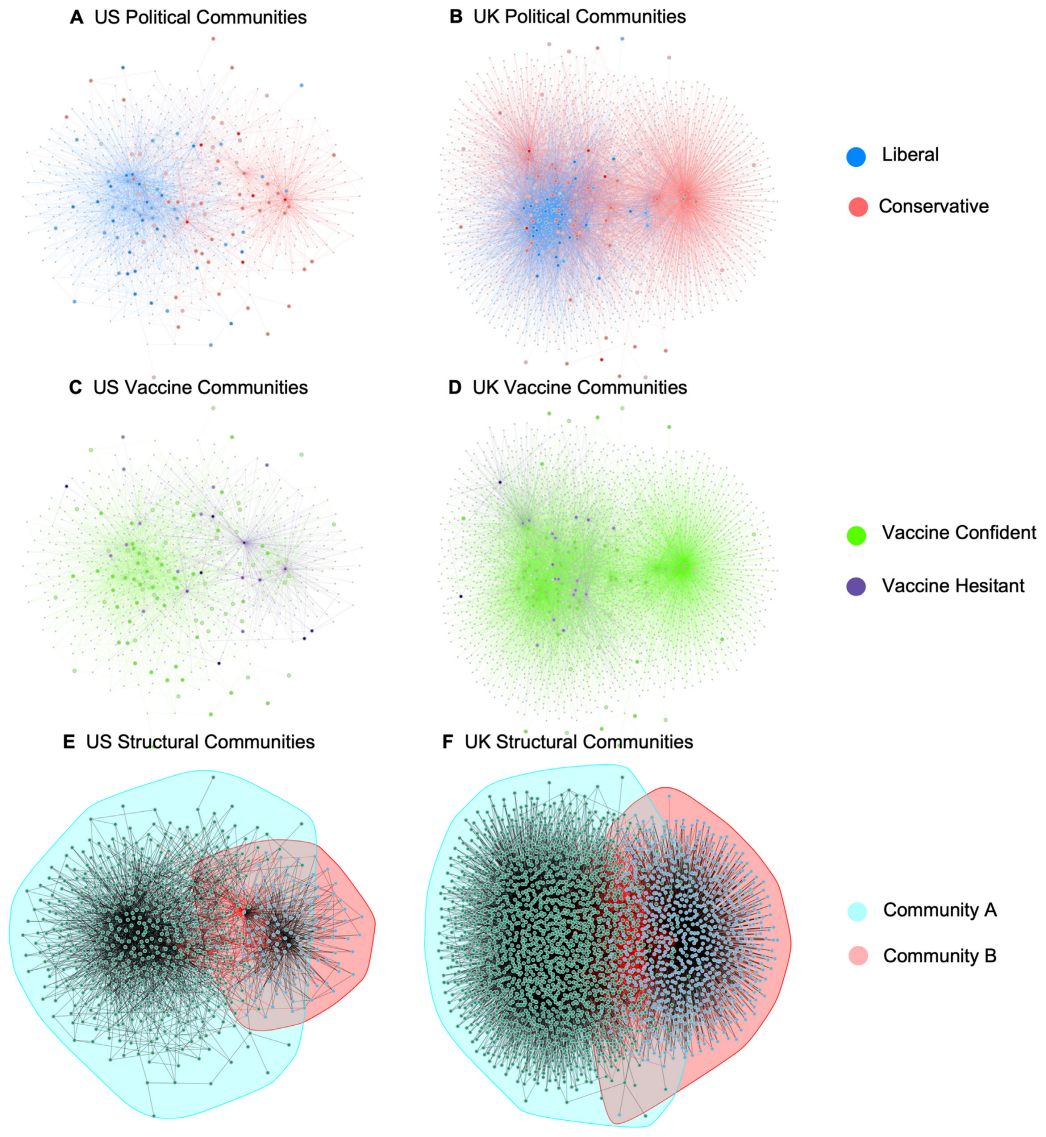
Visualizations of Twitter networks in the US (left) and UK (right). The first row shows the networks of those who are more liberal/left-wing (blue nodes) versus those who are more conservative/right-wing (red nodes) in the US (A) and UK (B). The second row shows the networks of vaccine confident individuals (green nodes) and vaccine hesitant individuals (purple nodes) in the US (C) and UK (D). In the third row, borders are drawn around structural communities identified by a label-propagation graph partitioning algorithm in the US (E) and UK (F). Each small uncolored node represents an influencer that at least three of the participants were following, and each large colored node represents a participant who is following an influencer. Each edge between two nodes represents a following relationship (one person following another Twitter account). Layouts of the graphs were created using the large-graph-layout algorithm to visually highlight community structures. Absolute distances between nodes are not meaningful in these visualizations.

To test whether low- and high-vaccine-confident individuals would cluster into “echo chambers” (H3), we conducted social network analyses. Specifically, we investigated whether participants and the influencers they followed clustered into structurally separate communities, and whether these structural separations corresponded with participants’ beliefs about politics and the vaccine. In Figure   1, we visualized the Twitter networks of the US and the UK with nodes colored based on political ideology (Panels A and B), with nodes colored based on vaccine attitudes (Panels C and D), and then automatically identified structural communities using a label-propagation graph partitioning method in the US and UK (Figure 1E and F). Detailed methods are in the “Materials and methods” Section.

We performed community detection analysis using a label-propagation algorithm that does not require prespecifying the number of communities (36). The algorithm identified two distinct structural communities (community A and community B; see Figure 1E and F). We calculated the average political conservatism and vaccine confidence of participants in each community. Here, we excluded “influencers,” or the accounts participants followed (which are plotted visually in Figure 1) since we do not have information about their political ideologies or vaccine opinions. In the US, the average political conservatism of participants in community B (6.07, 95% CI = [5.80, 6.35]) was far higher than that of participants in community A (3.43, 95% CI = [3.00, 3.86]), *t*(91.64) = 10.51, *d* = 2.20, *P* < 0.001. Additionally, in the US, the average vaccine confidence of participants in community A (5.98, 95% CI = [5.72, 6.24]) was also much higher than that of participants in community B (4.46, 95% CI = [3.45, 5.47]), *t*(15.05) = 3.12, *d* = 1.61, *P* = 0.007. In the UK, the average political conservatism of participants in community B (5.09, 95% CI = [4.33, 5.85]) was marginally higher than the political conservatism of community A (4.10, 95% CI = [4.10, 4.72]), *t*(14.62) = 1.80, *d* = 0.94, *P* = 0.092. However, the average vaccine confidence of community B (6.50, 95% CI [6.24, 6.76]) was higher than the average vaccine confidence of community A (6.06, 95% CI [5.87, 6.25]), *t*(26.52) = 2.92, *d* = 1.13, *P* = 0.007. In other words, in the US, participants in community B were more conservative and less vaccine-confident than participants in community A. In the UK, participants in community B were marginally more conservative and significantly more vaccine-confident than participants in community A.

Next, we investigated whether degree centrality (37) within the “liberal” community (community A) and the “conservative” community (community B) correlated with vaccine confidence. The degree centrality of a node refers to the number of links connected to the node, and can be used as an intuitive measure of how “central” a node is in a community. Although degree centrality was calculated for every node including both participants and the “influencers” they follow, the “influencers” were excluded from this analysis again since we do not have information about their vaccine and political attitudes. In the US, participants’ centrality within the “liberal” community was not significantly correlated with their vaccine confidence (*r* = 0.16, 95% CI [−0.03, 0.33], *P* = 0.402), whereas participants’ centrality within the “conservative” community was negatively correlated with their vaccine confidence (*r* = −0.22, 95% CI [−0.39, −0.03], *P* = 0.024). In the UK, however, participants’ centrality within the liberal community was not correlated with their vaccine confidence (*r* = 0.07, 95% CI [−0.12, 0.24], *P* = 0.480), nor was participants’ centrality within the conservative community (*r* = 0.15, 95% CI [−0.04, 0.32], *P* = 0.116). Thus, we find evidence that participants’ level of connectedness within the conservative community is negatively associated with participants having lower vaccine confidence in the US, but the patterns are less clear in the UK.

We performed robustness checks with different exclusion criteria, different ways of constructing the network graphs, and different network centrality statistics which, along with other network statistics such as average pathlength (38), modularity (39), and assortativity (40) are reported in Supplementary Appendix Material Section S2 and Supplementary Appendix Table S5 to S11; these results broadly support the idea that the US network was less connected than the UK network. We performed an additional network regression analysis to investigate potential associations between network structures and participant attitudes toward politics or vaccines; detailed methods and results are reported in the Supplementary Appendix Section S2, which generally show that political opinion divides are associated with network structural divides in the US but not in the UK.

### Specific influencers associated with vaccine confidence

To have a more granular picture of the kinds of Twitter “influencers” our participants followed, we explored some of the specific influencers in each community who had followers who were highest and lowest in vaccine hesitancy. For this analysis, we looked at Twitter influencers who were followed by at least 10 people from sample 1, and calculated their followers’ average vaccine confidence, as well as the proportion of followers in our sample who intended to or had received the vaccine.

**Fig. 2.**
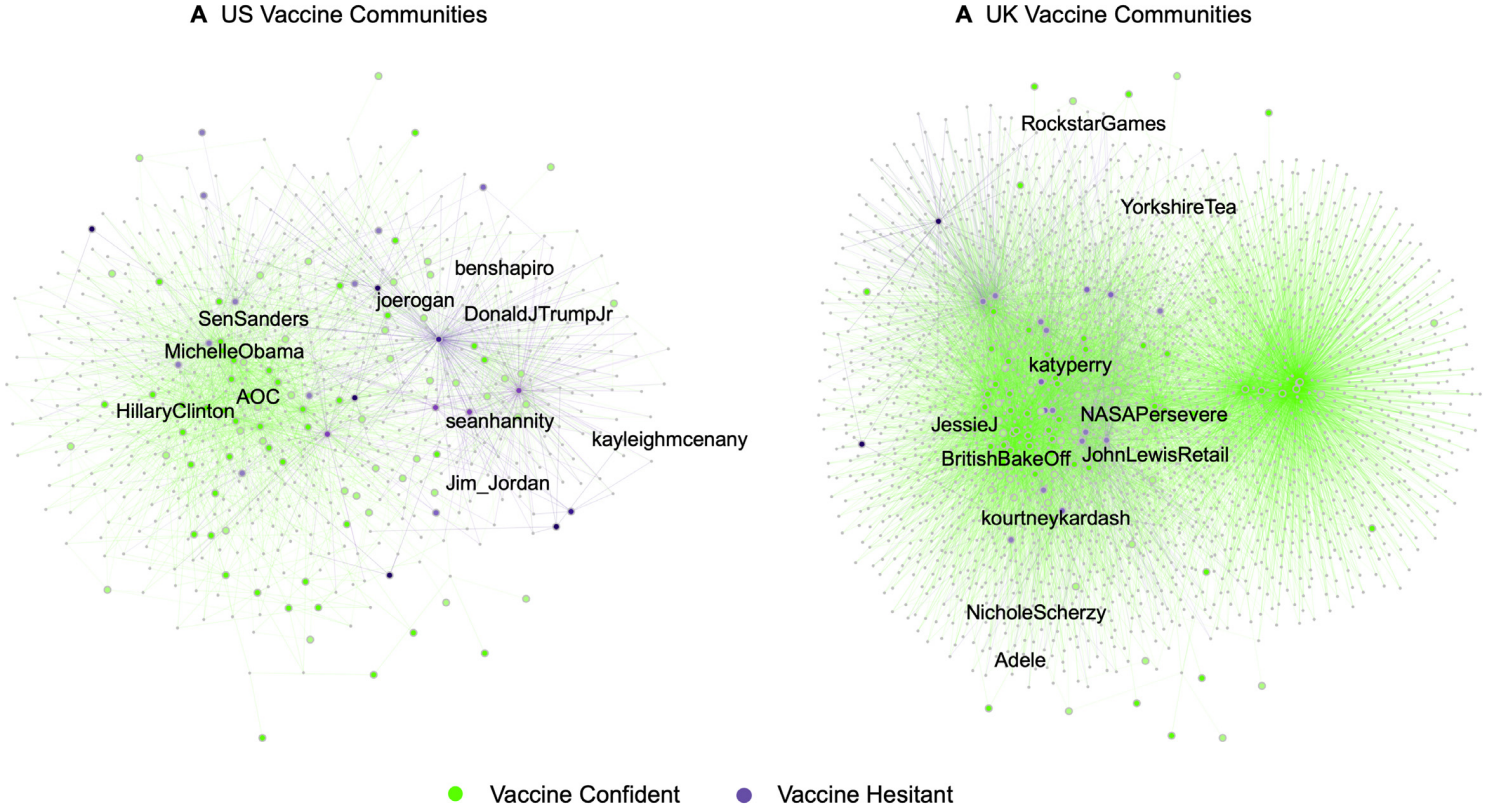
The same network plot from Figure 1 with example nodes (from Table 1) labeled. As shown above, more conservative influencers (e.g. Ben Shapiro, Sean Hannity) had followers who were lower in vaccine confidence (shaded purple), whereas more liberal influencers (e.g. Hillary Clinton, Alexandria Ocasio-Cortez) had followers who were higher in vaccine confidence (shaded green) in the US (Panel A). These patterns were less clear in the UK (Panel B).

The top 15 influencers associated with the highest and lowest vaccine confidence in the US and the UK, along with their membership in each community, are shown in Table 1 and plotted visually in Figure 2. In the US, right-leaning media personalities (e.g. Candace Owens, Ben Shapiro), Republican Party politicians (e.g. Senator Rand Paul), hyper-partisan news sources (e.g. Breaking 911), and a popular podcast host known for expressing vaccine hesitancy (Joe Rogan) (41), were among the top accounts associated with low vaccine confidence. By contrast, liberal/Democratic Party politicians (e.g. former Secretary of State Hillary Clinton, Congresswoman Alexandria Ocasio-Cortez) and left-leaning media sources (The Washington Post) were associated with high vaccine confidence. In the UK, on the other hand, vaccine confidence did not appear to be as politicized, and no clear patterns emerged. In the US, most of the top influencers associated with low vaccine confidence were primarily in the “conservative” community (community B), whereas most of the top influencers associated with high vaccine confidence were in the “liberal” community (community A). See Supplementary Appendix Tables S12 and 13 for robustness checks of this analysis using different thresholds.

**Table 1.** Twitter influencers associated with high and low vaccine confidence among their followers in the US and UK.

US				UK			
Twitter handle	Vaccine confidence	% Getting vaccine	Community membership	Twitter handle	Vaccine confidence	% Getting vaccine	Community membership
**Influencers associated with low vaccine confidence**							
RealCandaceO	3.15 (2.27)	30.00 (48.30)	B	Charlottegshore	4.10 (1.07)	40.00 (51.64)	B
joerogan	3.32 (2.11)	27.27 (46.71)	A	selenagomez	4.18 (1.37)	63.64 (50.45)	B
kayleighmcenany	3.55 (1.74)	30.00 (48.30)	B	example	4.25 (1.16)	80.00 (42.16)	A
TheBabylonBee	3.73 (2.34)	27.27 (46.71)	B	coldplay	4.36 (1.42)	72.73 (46.71)	A
dbongino	3.83 (1.91)	33.33 (49.24)	B	TheXFactor	4.40 (1.66)	40.00 (51.64)	A
benshapiro	3.92 (2.43)	50.00 (52.22)	B	LanaDelRey	4.45 (1.21)	72.73 (46.71)	B
RandPaul	3.95 (2.19)	40.00 (51.64)	B	RockstarGames	4.45 (1.47)	27.27 (46.71)	A
DonaldJTrumpJr	3.96 (1.80)	38.46 (50.64)	B	lilyallen	4.50 (1.60)	70.00 (48.30)	B
TuckerCarlson	4.00 (1.97)	42.86 (51.36)	B	JessieJ	4.57 (1.35)	60.00 (50.71)	A
seanhannity	4.04 (1.89)	38.46 (50.64)	B	NicoleScherzy	4.57 (1.40)	57.14 (51.36)	A
Jim_Jordan	4.05 (2.05)	45.45 (52.22)	B	Adele	4.57 (1.51)	73.33 (45.77)	A
JudgeJeanine	4.05 (2.05)	45.45 (52.22)	B	kourtneykardash	4.59 (1.20)	54.55 (52.22)	A
PressSec45	4.08 (2.22)	50.00 (52.22)	B	Drake	4.61 (1.33)	50.00 (51.89)	B
marklevinshow	4.15 (1.86)	40.00 (51.64)	B	katyperry	4.62 (1.40)	71.43 (46.29)	A
Breaking911	4.20 (2.15)	50.00 (52.70)	A	rihanna	4.63 (1.40)	69.57 (47.05)	A
**Influencers associated with high vaccine confidence**							
VP	6.62 (0.48)	100.00 (0.00)	A	sarapascoe	6.42 (0.42)	100.00 (0.00)	A
HillaryClinton	6.57 (0.65)	92.86 (26.73)	A	StephenMangan	6.36 (0.60)	100.00 (0.00)	A
MichelleObama	6.46 (0.54)	91.67 (28.87)	A	BarristerSecret	6.36 (0.55)	100.00 (0.00)	A
WhiteHouse	6.42 (0.97)	83.33 (38.92)	A	Misskeeleyhawes	6.35 (0.71)	100.00 (0.00)	B
ewarren	6.36 (0.74)	90.91 (30.15)	A	mrjamesob	6.35 (0.58)	100.00 (0.00)	A
KamalaHarris	6.35 (0.97)	92.31 (27.74)	A	Number10cat	6.33 (0.78)	100.00 (0.00)	A
AOC	6.31 (0.98)	90.48 (30.08)	A	Dawn_French	6.29 (0.72)	100.00 (0.00)	A
dog_feelings	6.23 (1.17)	81.82 (40.45)	A	richardosman	6.24 (0.77)	96.00 (20.00)	A
TheOnion	6.23 (0.73)	84.62 (37.55)	A	BritishBakeOff	6.23 (0.68)	90.91 (30.15)	A
washingtonpost	6.20 (1.01)	80.00 (42.16)	A	joelycett	6.22 (0.66)	100.00 (0.00)	A
SenSanders	6.17 (1.64)	83.33 (38.92)	A	NASAPersevere	6.21 (0.86)	100.00 (0.00)	A
POTUS	6.08 (1.51)	90.00 (30.78)	A	neiltyson	6.21 (0.80)	100.00 (0.00)	A
dog_rates	6.05 (1.75)	81.82 (40.45)	A	JohnLewisRetail	6.19 (0.97)	92.31 (27.74)	A
BarackObama	5.96 (1.37)	85.71 (35.63)	A	BootstrapCook	6.19 (0.93)	100.00 (0.00)	A
ActuallyNPH	5.91 (1.71)	81.82 (40.45)	A	RobertDyas	6.18 (1.10)	81.82 (40.45)	B

Note: The Twitter accounts associated with the top 15 highest and top 15 lowest mean vaccine confidence scores among their followers (1 = low confidence, 7 = high confidence) in the US and the UK are shown above, along with the percentage of followers who are or intend to get vaccinated. Standard deviations are shown in parentheses. Additionally, each influencer's community membership (generated via community detection analysis) is shown. Individuals with high self-reported vaccine confidence among their followers tend to reside in the “liberal” community (Community A), whereas individuals low in self-reported vaccine-confidence among their followers tend to reside in the “conservative” community (Community B). Only influencers who were followed by at least 10 participants in our datasets are shown in the above analysis. A total of 306 influencers were followed by at least 10 people in the US, and 492 influencers were followed by at least 10 people in the UK.

### Survey data

The politicization of vaccine attitudes in the US, but not the UK, was also seen in our survey data alone. Looking just at the survey data (using the full sample without Twitter handles), we found that self-reported political conservatism was negatively associated with vaccine confidence, *r* = −0.33, 95% CI = [−0.39, −0.26], *P* < 0.001. This relationship was present in both the US dataset, *r* = −0.43, 95% CI [−0.52, −0.35], *P* < 0.001, and, albeit weaker, in the UK dataset, *r* = −0.13, 95% CI [−0.24, −0.02], *P* = 0.026. The relationship between political conservatism and vaccine hesitancy was moderated by country (UK vs. US), *β* = −0.33, 95% CI = [−0.40, −0.25], *P* < 0.001, illustrating that vaccine confidence was more politically polarized in the US than the UK.

## Study 2

The aim of Study 2 was to expand on the findings of Study 1 by examining how self-reported vaccine hesitancy was associated with sharing and interacting with low-quality information in a larger sample (H4). We recruited a convenience sample of participants who had used the web application “Have I Shared Fake News.” (A link to the current version of the app is here: https://newsfeedback.shinyapps.io/HaveISharedFakeNews/.) Data collection for this app started in 2021 April, and ended 2021 October (for the purposes of this analysis), with most participants using the app in May and June of 2021 (see Supplementary Appendix Figure S3 for a full timeline). At the time of analysis, 6,727 people used the app and 2,359 provided Twitter handles. After excluding participants who followed more than 50,000 people (as these Twitter accounts were likely people entering the handles of public figures) and people who did not answer the vaccine likelihood question, we were left with a total sample size of 1,600 participants (749 M, 619 F, 33 non-binary/transgender/other, 199 No Response, *M*_age_ = 38.4, SD_age_ = 12.6). Since we also invited Study 1 participants to use this app, 195 participants in this dataset were overlapping with the Study 1 participants. Location data were not collected via this app, meaning we could not explore differences between countries.

When using this app, participants who consented to take part in research were asked “How likely are you to get vaccinated for COVID-19 when it becomes available?” on a 1 to 100 scale (0 = very unlikely and 100 = very likely) (*M* = 93.36, SD = 21.55). A total of 242 participants reported a vaccine likelihood score of less than 100, and 93 participants reported a score of less than 50. People also entered demographic information in exchange for information about their news sharing behavior on Twitter. For example, people entered their political orientation on a 7-point scale (1 = “Extremely Liberal”; 7 = “Extremely Conservative”) (*M* = 2.64, SD = 1.50). We also measured a number of other variables through this app, such as political conservatism, affective polarization (favorability toward the ingroup minus favorability toward the outgroup) (42), conspiracy mentality (43), mental health, life satisfaction (43), age, gender, and having a Bachelor's degree, which we used as additional control variables in our regression models. See Supplementary Appendix S1 for full question wording. Since this was a convenience sample, it was more left-leaning and contained more vaccine-confident participants. Thus, while it was not ideal for visualizing network plots (because the “liberal” and “vaccine-confident” networks would be quite large, and many users of the app followed each other), it provided a larger sample of participants to examine associations between interacting with online misinformation and vaccine hesitancy.

### Engagement with news on social media and vaccine confidence

We tested whether one's self-reported likelihood of receiving the vaccine predicted sharing or interacting with lower-quality information online. To do this, we first examined whether one's likelihood of receiving the COVID-19 vaccine was associated with the number of hyper-partisan URLs one shared on their Twitter timeline, based on a prior list of hyper-partisan URLs (32) and the “Iffy News” Index (44). This variable was collected at the time participants used the app and shown to participants as part of their “fake news score.” A total of 1030 hyper-partisan websites were shared in the full sample, and people shared about 0.77 (SD = 5.34) hyper-partisan news URLs on their Twitter timeline on average. One's likelihood of receiving the vaccine negatively predicted the sharing of hyper-partisan news sites, *β* = −0.07, 95% CI = [−0.12, −0.02], *P* = 0.007. This effect remained significant when controlling for a number of other factors measured in the app, such as political conservatism, affective polarization (favorability toward the ingroup minus favorability toward the outgroup), conspiracy mentality, mental health, life satisfaction, age, gender, having a Bachelor's degree, number of followers, and number of accounts followed, *β* = −0.14, 95% CI = [−0.20, −0.07], *P* < 0.001. Indeed, in this multiple regression, the only remaining significant predictor of hyper-partisan news sharing besides vaccine hesitancy was age, *β* = 0.21, 95% CI = [0.14, 0.29], *P* < 0.001, replicating prior work about age and fake news sharing (27), and affective polarization was a marginally significant predictor, *β* = 0.08, 95% CI = [0.00, 0.16], *P* = 0.053 (all other *P*s > 0.184). Overall, these results support H4. See Supplementary Appendix Tables S14 and 15 for full regression models and robustness checks.

We then replicated the above analysis using a more sensitive measure of the quality of news shared. To do this, we used *NewsGuard*, which provided us with a dataset of over 4500 news URLs along with a trustworthiness rating of each URL (as an example, *breitbart.com* has a trustworthiness rating of 49.5 out of 100). Using the Twitter API, we scraped 1,831,308 tweets from timelines of 1600 participants using the Twitter handles participants provided when they used the app. Of these, 46,202 contained URLs that could be given a “trustworthiness” rating by *NewsGuard*. We calculated a variable indicating the average trustworthiness of URLs shared per user. The mean trustworthiness of the URLs participants shared was 92.88 (SD = 14.01), indicating that our sample tended to share trustworthy news.

**Fig. 3.**
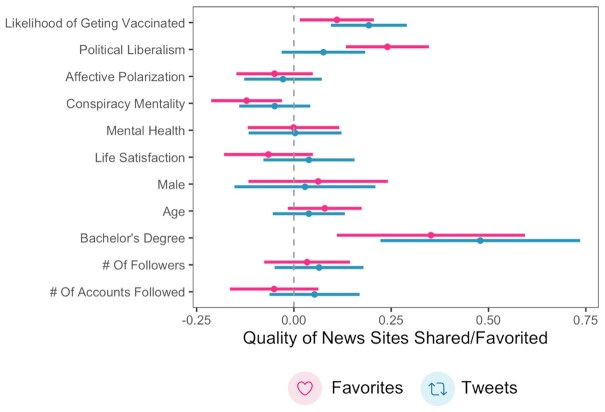
One's self-reported likelihood of getting the COVID-19 vaccine predicted the overall quality of news participants shared publicly (tweeted) or liked (favorited). These associations remained significant in multiple regression analyses accounting for political liberalism, affective polarization, conspiracy mentality, mental health, life satisfaction, age, gender, education, number of followers, and number of accounts followed. Error bars represent 95% confidence intervals, and standardized beta coefficients are shown for ease of interpretation.

Again, one's likelihood of receiving the vaccine predicted the quality of news URLs people shared online, *β* = 0.23, 95% CI = [0.17, 0.29], *P* < 0.001. This effect remained significant when including all relevant control variables, *β* = 0.19, 95% CI = [0.09, 0.29], *P* < 0.001. The only other significant predictor of the quality of news people shared besides vaccine confidence in this model was having a Bachelor's degree, *β* = 0.48, 95% CI = [0.22, 0.74], *P* < 0.001 (all other *Ps* > 0.166). This model is plotted in Figure 3.

Finally, given that different types of content on social media tend to receive retweets as opposed to favorites (or likes) (30)—possibly because retweets are more public than favorites—we then examined whether one's likelihood of receiving the vaccine predicted favorites as well. We scraped 1,876,635 favorites from our sample, 61,140 of which contained URLs that could be given a *NewsGuard* “trustworthiness” rating (*M* = 93.59, SD = 12.84). One's likelihood of getting vaccinated once again predicted favoriting higher-quality news, with a similar effect size, *β* = 0.23, 95% CI = [0.06, 0.23], *P* < 0.023. Once again, this effect remained significant when accounting for all control variables, *β* = 0.11, 95% CI = [0.02, 0.21], *P* = 0.023. The other significant predictors of favoriting low-quality news in this model were political liberalism, *β* = 0.24, 95% CI = [0.13, 0.35], *P* < 0.001; conspiracy mentality, *β* = −0.12, 95% CI = [−0.21, −0.03], *P* = 0.009; and having a Bachelor's degree, *β* = 0.35, 95% CI = [0.11, 0.59], *P* = 0.004. We also found that the quality of news URLs participants shared correlated strongly with the quality of news URLs that they favorited, *r* = 0.53, 95% CI = [0.47, 0.58], *P* < 0.001. Thus, an individual's more public sharing behavior online may not be strongly different than their more private favoriting behavior. Interestingly, across all three models, vaccine hesitancy was a robust predictor of interacting with online misinformation, whereas other variables such as age, political ideology, conspiracy mentality, and education were more inconsistent predictors.

### Specific news sites associated with vaccine hesitancy

For a more granular examination of this data, we explored the specific news sites that tended to be shared by those who were less likely to receive the vaccine. To do this, we examined URLs that were shared by at least 10 people in the dataset and calculated the average likelihood of getting the vaccine among these news sharers. We also examined news sites favorited by at least 10 people in the dataset and calculated their average likelihood of getting the vaccine. Several news sites shared and favorited by those who reported being unlikely to get the vaccine (e.g. “zerohedge.com,” “palmerreport.com,” “breitbart.com,” “rt.com,” “thefederalist.com,” “tmz.com”) are rated as “untrustworthy” by *NewsGuard* (see Table 2). See Supplementary Appendix Tables S16 and 17 for robustness checks of this analysis using different thresholds.

**Table 2.** Specific URLs shared or favorited associated with low self-reported likelihood of receiving the vaccine.

News website shared	Likelihood of getting vaccine	News website favorited	Likelihood of getting vaccine
billboard.com	69.08 (42.21)	billboard.com	70.07 (41.76)
zerohedge.com	72.62 (42.52)	deviantart.com	72.30 (41.00)
foxbusiness.com	73.50 (41.05)	zerohedge.com	73.94 (39.67)
washingtonexaminer.com	75.92 (35.16)	thepostmillennial.com	75.23 (38.98)
upworthy.com	79.15 (32.80)	thewrap.com	77.73 (38.93)
express.co.uk	80.69 (38.57)	breitbart.com	78.07 (37.21)
thefederalist.com	82.00 (38.24)	dailycaller.com	79.25 (38.59)
thinkprogress.org	83.46 (36.37)	rt.com	80.83 (33.67)
money.cnn.com	83.94 (32.36)	abc13.com	82.23 (37.41)
boston.com	84.10 (34.70)	webmd.com	83.18 (37.57)
tmz.com	85.38 (35.73)	tabletmag.com	84.47 (34.86)
gq.com	85.43 (32.15)	apod.nasa.gov	84.55 (33.28)
courier-journal.com	86.20 (32.56)	palmerreport.com	85.64 (26.18)
bizjournals.com	87.55 (28.10)	heraldscotland.com	86.06 (29.13)
spiegel.de	87.89 (29.13)	foxnews.com	88.21 (30.62)

Note: On the left are the URLs that are tweeted by at least 10 people along with the average likelihood of getting the vaccine (on a scale from 1 to 100) among those that tweeted each URL. On the right are the URLs that were favorited by at least 10 people along with the average likelihood of getting the vaccine among those that favorited each URL. Several of the news sites shown (e.g. “zerohedge.com,” “palmerreport.com,” “breitbart.com,” “rt.com,” “thefederalist.com,” “tmz.com”) received low trustworthiness ratings by *NewsGuard*.

## Discussion

Across two studies with unique datasets connecting survey data about self-reported vaccine confidence to social media data, we found that social media behavior is associated with attitudes about the vaccine. Specifically, following US Republican Twitter influencers and hyper-partisan or low-quality news sites negatively predicted confidence in the COVID-19 vaccine, though following politicians from the UK's Conservative party did not predict confidence in the vaccine. These results held even when controlling for a number of relevant variables, such as self-reported ideology and education, meaning that social media behavior explains unique variance in predicting vaccine attitudes.

Community detection analysis revealed that Twitter networks in the US and the UK are divided into structural communities (or “echo chambers”) broadly reflecting liberal and conservative attitudes. Centrality in the more “conservative” community in the US negatively predicted self-reported vaccine confidence; however, this was not true in the UK. A specific examination of the influencers in each cluster found that prominent US influencers associated with the Republican party (e.g. Tucker Carlson, Candace Owens), as well as influencers who have caused controversy about spreading misinformation about the vaccine online (e.g. Joe Rogan) (41) tended to have followers with low levels of vaccine confidence.

Finally, in Study 2, we found that one's likelihood of receiving the vaccine was associated with the quality of news articles shared (tweeted) and liked (favorited) on Twitter, even when controlling for demographic variables. This suggests that vaccine-hesitant individuals are not only consuming lower-quality news, but are spreading lower-quality news to their networks. These results were similar when looking at both more private forms of engagement (favorites) and more public forms of social media sharing (retweeting), which were highly correlated with each other.

One limitation of this work is that it captures a specific time-point in history. Most of the data were collected around the summer of 2021, and dynamics around these issues online and offline may have evolved. Furthermore, there are limitations with our samples. Neither study was nationally representative, though Study 1 was roughly politically-balanced and included a large portion of vaccine-hesitant and vaccine-neutral respondents. Study 2, while larger and better-powered to analyze the amount of misinformation shared by individuals, was a convenience sample recruited online via an app. It is possible that some of our findings were influenced by idiosyncrasies of these two samples. That said, it's important to note that the main conclusions were consistent across both samples

An important limitation of this work is that it is correlational. While our results are consistent with the theory that exposure to misinformation and partisan cues in one's online social network influences vaccine attitudes, they are also consistent with other interpretations, such as vaccine-hesitant individuals selectively following and engaging with content that confirms their beliefs (24, 45). Twitter following behavior could also be a proxy for other kinds of media exposure (for instance, people who follow Republican politicians may also frequently watch Fox News). Other research should follow up on this study by testing the causal effects of exposure to certain information sources on vaccine attitudes, through lab and field experiments, network interventions that manipulate the structure of one's network (46), or network modeling approaches (47).

Many of the effect sizes we found were small-to-medium (e.g. *r* = 0.23 for the correlation between quality of news shared and likelihood of getting the vaccine) (48, 49), though other effect sizes were large, such as the difference in vaccine confidence between participants in the liberal community (community A) and the conservative community (community B) in the network. Since almost 4 billion people use social media worldwide (50), even small associations between exposure to certain types of online content and vaccine beliefs are practically significant.

There are also multiple possible reasons for differences between the UK and US samples. For instance, they may reflect differences in conservatism between the US and the UK. It has been noted that the UK conservatives are generally less conservative than conservatives in the US, or that UK conservatism may reflect different priorities and values, such as traditionalism (51). Though, another interpretation behind the differences we found in the US and the UK is that partisan elite cues early in the pandemic guided polarization around the vaccine, and certain political figures, such as Donald Trump or Conservative Prime Minister Boris Johnson, played an important role in driving opinions about COVID-19 early on. Indeed, Conservative Prime Minister Boris Johnson called antivaxxers “nuts” in 2020 (52). By contrast, one study from 2020 estimated that Donald Trump was the largest source of COVID-19 misinformation at the time (53). Experiments support the idea that partisan elite cues play a causal role in sharing opinions about the virus (17, 54, 55).

Our results demonstrate potential challenges of promoting vaccine confidence in a polarized social media environment (56–58), since accurate messages about the vaccine may not be seen by those who need it most unless they come from trusted influencers in their networks, such as influencers associated with the Republican party. Hopefully, these results will help researchers and policymakers understand and help create solutions for vaccine hesitancy. For example, targeted messages from figures trusted by people in communities associated with low vaccine confidence (17, 59), interventions that protect against susceptibility to misinformation (60–63) or algorithmic solutions that improve the overall quality of news presented to people on social media (35) may be useful for improving vaccine confidence. Amid frequent discussion about an “infodemic” of misinformation on social media contributing to vaccine hesitancy (5) and controversy over prominent influencers such as Joe Rogan spreading vaccine misinformation online (41), our work demonstrates the crucial link between online behavior and vaccine attitudes.

## Materials and methods

Code, surveys, materials, dictionaries, lists of URLs used, and de-identified data are available at: https://osf.io/shjdb/. We could not share all Twitter data due to privacy concerns (e.g. Twitter handles, raw Twitter texts, or raw URLs shared), though we share limited, anonymized data and code for replicating the main models and network analysis. Furthermore, lists of URLs and Twitter handles along with their “trustworthiness” ratings cannot be accessed without a license agreement from *NewsGuard. NewsGuard* data were accessed on 2022 February 29, and reflects ratings as of that particular date. Data were analyzed using R version 4.0.1. The study was preregistered at: https://aspredicted.org/blind.php?x = c2jx6q. This study was approved by the University of Cambridge Psychology Research Ethics Committee (PRE.2020.144).

We made minor deviations from our preregistration. First, we did not focus on associations between the misinformation susceptibility test (64), life satisfaction, mental health, and Twitter behavior (except for briefly in Study 2), since they will be the focus of future publications and are not as relevant to the current work. Second, we said that we would examine influencers who are followed by at least 25 participants, following (30), and calculate the average vaccine attitudes of their followers. Because we had a smaller sample of vaccine-hesitant participants than anticipated, we instead used a threshold of 10. However, we show the results from this same analysis using different thresholds in Supplementary Appendix Material S12 and 13, finding qualitatively similar results (e.g. following conservative influencers in the US seems to be associated with vaccine hesitancy across multiple thresholds).

### Participants

For Study 1, we collected a total sample of 1,246 participants (465 M, 556 F, 15 non-binary/transgender/Other, *M*_age_ = 44.33) via the survey platform Prolific Academic from 2021 May 11 to 2021 June 29. To recruit a large enough sample of vaccine-hesitant participants, as well as politically diverse participants, we used Prolific prescreening criteria to recruit a target sample size of 400 participants who reported being either hesitant or neutral about the COVID-19 vaccine. In addition to this, we recruited 200 US liberals, 200 US conservatives, 200 UK liberals, and 200 UK conservatives. This is a slight deviation from the preregistration, where we said we would sample 300 conservative politicians and 300 liberal politicians, but did not mention anything about the country. Because we were interested in the dynamics of vaccine hesitancy in multiple countries, we decided to collect a slightly larger sample of liberals and conservatives from the US and the UK. A total of 587 participants voluntarily provided their Twitter handles, of which we were able to scrape 464 follower networks for analysis (175 M 210 F, 6 transgender/non-binary/other, 73 Missing; *M*_age_ = 37.7; SD = 12.5). In addition to our key measures, we asked a number of other measures as well, such as a measure of misinformation susceptibility (64), mental health, life satisfaction, country, and education. We report other demographic data in Supplementary Appendix Table S1.

For Study 2, we recruited a convenience sample of participants who had used the web application “Have I Shared Fake News.” We shared the web application on Twitter in 2021 May, and recruited participants up until 2021 October via snowball sampling. We also gave Study 1 participants the opportunity to use the app. While some of this dataset was collected before the preregistration, much of it was collected afterward as well, and it was not analyzed until after the preregistration. See Supplementary Materials Figure S2 for more information about when the dataset was collected.

### Network analysis

We constructed community network graphs for the US and the UK participants and the “influencers” they follow (that are followed by at least 3 participants). Before filtering out small influencers, the dataset contained in total 50,276 following relationships from 124 participants in the US and 77,160 following relationships from 123 participants in the UK. The smaller number of participants in both countries is the result of filtering out participants who did not report political conservatism or vaccine confidence values, both of which are necessary for the network analysis. After filtering out influencers who were not followed by at least three participants, we constructed network graphs based on the 2,588 following relationships from 109 participants in the US, and the 11,055 following relationships from 118 participants in the UK. See Supplementary Appendix Section S2 for further explanation about the different number of following relationships in the US and the UK and a robustness check of the filtering criteria for influencers. After constructing the network graphs, we calculated several descriptive statistics with the main “Complete Networks” that included influencers and modularity coefficients and assortativity coefficients with “Commonality Networks” that excluded influencers, see Supplementary Table S5.

Then, we used a label-propagation algorithm for graph partitioning (35) to identify two structural communities in the US and the UK. We chose the label-propagation algorithm because it was designed for large-scale complex networks and can be performed at near linear-time, which is suitable for our network dataset and limited computational power. We calculated and compared the average political and vaccine attitudes among participants within each community in the US and the UK. We did not include influencers in this comparison since their political and vaccine attitudes are unknown to us and cannot be reliably interpolated. To examine the relationship between structural properties of the network and attitude differences, we correlated the Degree Centrality of a node in a community with a node's political or vaccine opinion. We also performed network regression between the structural distance matrices (i.e. the adjacency matrices) and the attitudinal distance matrices in terms of politics and vaccine opinions, following the method of Multiple Regression Quadratic Assignment Procedure with Double Semi Partialing (62). Influencers were also excluded in these analyses since we cannot reliably infer their vaccine opinion or political attitude. See Supplementary Appendix Section S2 for further explanation about the network analysis.

## Supplementary Material

pgac207_Supplemental_File

## Data Availability

Code, surveys, materials, dictionaries, lists of URLs used, and de-identified data are available at: https://osf.io/shjdb/. We could not share all Twitter data due to privacy concerns (e.g. Twitter handles, raw Twitter texts, or raw URLs shared), though we share limited, anonymized data and code for replicating the main models and network analysis. Furthermore, lists of URLs and Twitter handles along with their “trustworthiness” ratings cannot be accessed without a license agreement from *NewsGuard*.
