# Correction to: Social media behavior is associated with vaccine hesitancy

**DOI:** 10.1093/pnasnexus/pgad471

**Published:** 2024-01-30

This is a correction to: Steve Rathje, James K He, Jon Roozenbeek, Jay J Van Bavel, Sander van der Linden, Social media behavior is associated with vaccine hesitancy, *PNAS Nexus*, Volume 1, Issue 4, September 2022, pgac207, https://doi.org/10.1093/pnasnexus/pgac207

During a retroactive audit conducted by *PNAS Nexus*, it was discovered that this paper was missing a statement acknowledging compliance with the *PNAS Nexus* Human and Animal Participants and Clinical Trials policy:

All participants provided informed consent before answering surveys and providing their Twitter handles.

This error has been corrected in the original article.

