# Supplementary material for: Social media behavior is associated with vaccine hesitancy
Source: PNAS Nexus. 2022 Sep 30;1(4):pgac207. doi: 10.1093/pnasnexus/pgac207 (PMC9802475; doi:10.1093/pnasnexus/pgac207)
Supplement: pgac207_Supplemental_File [file pgac207_supplemental_file.docx]

**Supplementary Information for**

Social media behavior is associated with vaccine hesitancy

Steve Rathje^1^, James K. He^1^, Jon Roozenbeek^1^, Jay J. Van Bavel^2^, Sander van der Linden^2^

^1^Department of Psychology, University of Cambridge, Cambridge CB2 3RQ, United Kingdom

^2^ Department of Psychology & Center for Neural Science, New York University, New York, NY 10003

**Corresponding Author Emails:** sjr210@cam.ac.uk and sander.vanderlinden@psychol.cam.ac.uk

**Section S1:** Question Wording

Below we list the exact question wording for the survey questions participants were asked that are relevant to this study for Sample 1 and Sample 2.

Study 1:

*COVID-19 Vaccination Intentions:*

Have you or do you intend to receive the COVID-19 vaccine when you are eligible to do so?

● Yes, I have already received at least one dose of the COVID-19 vaccine

● Yes, I haven't received my first dose, but I intend to do so

● No, I haven't received my first dose and I am uncertain about whether I will get one

● No, I haven't received my first dose and I do not intend to get one

● I cannot get vaccinated against COVID-19 due to medical reasons

*COVID-19 Vaccine Safety & Efficacy:*

The currently available COVID-19 vaccines are…:

● Safe [1 “strongly disagree”, 7 “strongly agree]

● Effective in preventing the disease [1 “strongly disagree”, 7 “strongly agree]

*Political Ideology*

[United States]

Which of the following best describes your political preference?

● Extremely liberal

● Liberal

● Slightly liberal

● Moderate

● Slightly conservative

● Conservative

● Extremely conservative

[United Kingdom]

Which of the following best describes your political preference?

● Extremely left-wing/liberal

● Left-wing/liberal

● Slightly left-wing/liberal

● Middle of the road

● Slightly right-wing/conservative

● Right-wing/conservative

● Extremely right-wing/conservative

*Age:*

What is your year of birth?

*Gender:*

What is your gender?

- Male
- Female
- Transgender Female
- Transgender Male
- Trans/Non-Binary
- Not Listed
- Prefer not to Say

*Education:*

[United States]

What is the highest level of school you have completed or the highest degree you have received?

- Less than high school degree
- High school graduate (high school diploma or equivalent including GED)
- Some college but no degree
- Associate degree in college (2-year)
- Bachelor's degree in college (4-year)
- Master's degree
- Doctoral degree
- Professional degree (JD, MD)

[United Kingdom]

What is the highest level of education that you completed?

- No formal education above age 16
- Professional or technical qualifications above age 16
- School education up to age 18
- Degree (Bachelor's) or equivalent
- Degree (Master's) or other postgraduate qualification
- Doctorate

*US or UK:*Are you a resident of the United Kingdom or the United States?

*Country:*

In which country do you currently reside?

[drop-down list of countries]

Study 2:

*COVID-19 Vaccine Confidence:*

How likely are you to get vaccinated for COVID-19 when it becomes available? (0 = very unlikely and 100 = very likely)? If you have already received the vaccine, you may select 100.

*Political Orientation:*

What is your political orientation?

● Extremely liberal

● Liberal

● Slightly liberal

● Moderate

● Slightly conservative

● Conservative

● Extremely conservative

*Gender:*

What is your gender?

- Male
- Female
- Transgender Male
- Transgender Female
- Non-Binary/Other

*Age:*

How old are you?

*Education:*

What is the highest level of education you've completed?

- High School or Less
- Some College
- Bachelor’s Degree
- Higher Degree

Please choose whichever ethnicity that you identify with (you may choose more than one option):

- White/Caucasian
- Black or African American
- American Indian or Alaska Native
- Asian
- Native Hawaiian or Pacific Islander
- Other

*Favorability toward Democrats:*

How favorable do you feel toward Democrats (0 = very unfavorable and 100 = very favorable)?

*Favorability toward Republicans:*

How favorable do you feel toward Republicans (0 = very unfavorable and 100 = very favorable)?

*Life Satisfaction:*

In general, how satisfied are you with your life (0 = very unsatisfied and 100 = very satisfied)?

*Mental Health:*

In general, how would you rate your mental health? (0 = poor and 100 = very good)?

*Conspiracy Mentality:*

Some political and social events are debated, for example the 9/11 attacks, the death of Lady Diana, the assassination of John F. Kennedy. It is suggested that the ‘official version’ of these events could be an attempt to hide the truth to the public. What do you think about the following statement: I think that the official version of the events given by the authorities very often hides the truth (0 = completely false and 100 = completely true)?

**Table S1.** Demographics of Each Sample

| **Study 1 (Full Sample)** | | **Study 1 (Twitter Handles Only)** | | **Study 2 (App Dataset)** | |
| --- | --- | --- | --- | --- | --- |
| **(N=1246)** | | **(N=464)** | | **(N = 1600)** | |
| **Vaccine Confidence** | | **Vaccine Confidence** | | **Likelihood of Getting Vaccine** | |
| Mean (SD) | 5.34 (3.29) | Mean (SD) | 5.32 (1.49) | Mean (SD) | 93.3 (21.6) |
| Median [Min, Max] | 5.50 [1.00, 100] | Median [Min, Max] | 6.00 [1.00, 7.00] | Median [Min, Max] | 100 [0, 100] |
| Missing | 34 (2.7%) | Missing | 1 (0.2%) | **Age** | |
| **Will Get Vaccine** | | **Will Get Vaccine** | | Mean (SD) | 38.4 (12.6) |
| Will not get vaccine | 349 (27.3%) | Will not get vaccine | 118 (25.4%) | Median [Min, Max] | 36.0 [-53.0, 77.0] |
| Will get vaccine | 896 (70.0%) | Will get vaccine | 345 (74.4%) | Missing | 503 (31.4%) |
| Missing | 35 (2.7%) | Missing | 1 (0.2%) | **Political Conservatism** | |
| **Country** | | **Country** | | Mean (SD) | 2.64 (1.50) |
| UK | 548 (42.8%) | UK | 225 (48.5%) | Median [Min, Max] | 2.00 [1.00, 7.00] |
| US | 454 (35.5%) | US | 158 (34.1%) | Missing | 206 (12.9%) |
| Other/Missing | 278 (21.7%) | Other/Missing | 81 (17.6%) | **Gender** | |
| **UK or US** | | **UK or US** | | Male | 749 (46.8%) |
| United Kingdom | 281 (22.0%) | UK | 120 (25.9%) | Female | 619 (38.7%) |
| United States | 372 (29.1%) | US | 123 (26.5%) | Transgender Female | 2 (0.1%) |
| Neither | 15 (1.2%) | Other | 6 (1.3%) | Transgender Male | 1 (0.1%) |
| Missing | 612 (47.8%) | Missing | 215 (46.3%) | Non-Binary/Other | 30 (1.9%) |
| **Age** | | **Age** | | Missing | 199 (12.4%) |
| Mean (SD) | 34.9 (12.1) | Mean (SD) | 37.7 (12.5) | **Education** | |
| Median [Min, Max] | 32.0 [18.0, 73.0] | Median [Min, Max] | 36.0 [18.0, 73.0] | High School or Less | 69 (4.3%) |
| Missing | 251 (19.6%) | Missing | 74 (16.1%) | Some College | 218 (13.6%) |
| **Gender** | | **Gender** | | Bachelor's Degree | 421 (26.3%) |
| Male | 465 (36.3%) | Male | 175 (38.0%) | Higher Degree | 686 (42.9%) |
| Female | 556 (43.4%) | Female | 210 (45.3%) | Missing | 206 (12.9%) |
| Transgender Female | 3 (0.2%) | Transgender Female | 0 (0.0%) | **Ethnicity** | |
| Transgender Male | 9 (0.7%) | Transgender Male | 5 (1.1%) | White/Caucasian | 1148 (71.8%) |
| Non-Binary/Other | 2 (0.2%) | Non-Binary/Other | 1 (0.2%) | Black or African American | 33 (2.1%) |
| Prefer Not To Answer | 1 (0.1%) | Not Listed | 0 (0%) | American Indian or Alaska Native | 3 (0.2%) |
| Missing | 244 (19.1%) | Missing | 73 (15.8%) | Asian | 71 (4.4%) |
| **Political Conservatism** | | **Political Conservatism** | | Native Hawaiian or Pacific Islander | 3 (0.2%) |
| Mean (SD) | 3.97 (1.95) | Mean (SD) | 4.10 (1.93) | Other | 67 (4.2%) |
| Median [Min, Max] | 5.00 [1.00, 7.00] | Median [Min, Max] | 5.00 [1.00, 7.00] | Multiple Options Selected | 66 (4.1%) |
| Missing | 612 (47.8%) | Missing | 214 (46.4%) | Missing | 209 (13.1%) |
| **Bachelors** | | **Bachelors** | | **Followers** | |
| No Bachelor's Degree | 256 (20.0%) | No Bachelor's Degree | 99 (21.3%) | Mean (SD) | 1370 (3810) |
| Bachelor's Degree | 412 (32.2%) | Bachelor's Degree | 150 (32.3%) | Median [Min, Max] | 330 [0, 45900] |
| Missing | 612 (47.8%) | Missing | 215 (46.3%) | **# of Accounts Followed** | |
|  |  | **# of Accounts Followed** | | Mean (SD) | 1040 (1700) |
|  |  | Mean (SD) | 572 (1010) | Median [Min, Max] | 544 [0, 34300] |
|  |  | Median [Min, Max] | 189 [1.00, 5000] | **Number of Tweets** | |
|  |  |  |  | Mean (SD) | 2030 (1290) |
|  |  |  |  | Median [Min, Max] | 2840 [1.00, 3200] |
|  |  |  |  | **Number of Hyperpartisan Sites Shared** | |
|  |  |  |  | Mean (SD) | 0.767 (5.34) |
|  |  |  |  | Median [Min, Max] | 0 [0, 151] |

**Note**. Descriptive Statistics for all samples. Some descriptive statistics from Study 1 are missing because 1) these questions were added to the survey partway through data collection, or 2) participants chose not to answer certain questions. Because we had different questions assessing education in the US and the UK, we re-coded this particular variable to measure whether a participant had a bachelor’s degree for the purpose of presenting descriptives for the overall sample. Additionally, since we had two variables measuring country (a drop-down list versus a UK/US/other question), when subsetting the data for network analysis, we included participants who said US on either of the questions in the US dataset for network analysis, and included participants who said UK on either of the questions in the UK dataset for network analysis.

**Table S2.** Study 1 Regression Models

|  | Model 1 | Model 2 | Model 3 | Model 4 | Model 5 | Model 6 | Model 7 | Model 8 |
| --- | --- | --- | --- | --- | --- | --- | --- | --- |
| (Intercept) | 0.00 | 0.00 | 0.00 | 0.00 | -0.15 | -0.14 | -0.16 | -0.15 |
|  | [-0.09, 0.09] | [-0.09, 0.09] | [-0.09, 0.09] | [-0.09, 0.09] | [-0.38, 0.08] | [-0.38, 0.09] | [-0.39, 0.07] | [-0.38, 0.08] |
| Republicans | -0.12 * |  |  |  | -0.18 ** |  |  |  |
|  | [-0.21, -0.03] |  |  |  | [-0.30, -0.05] |  |  |  |
| Tory |  | 0.06 |  |  |  | 0.03 |  |  |
|  |  | [-0.04, 0.15] |  |  |  | [-0.10, 0.16] |  |  |
| HyperPartisan |  |  | -0.15 ** |  |  |  | -0.20 ** |  |
|  |  |  | [-0.24, -0.06] |  |  |  | [-0.32, -0.08] |  |
| BadHandles |  |  |  | -0.19 *** |  |  |  | -0.19 ** |
|  |  |  |  | [-0.28, -0.10] |  |  |  | [-0.32, -0.06] |
| Politics |  |  |  |  | -0.30 *** | -0.32 *** | -0.29 *** | -0.29 *** |
|  |  |  |  |  | [-0.43, -0.17] | [-0.45, -0.19] | [-0.42, -0.17] | [-0.42, -0.16] |
| Age |  |  |  |  | 0.11 | 0.09 | 0.11 | 0.13 |
|  |  |  |  |  | [-0.02, 0.24] | [-0.04, 0.22] | [-0.02, 0.24] | [-0.01, 0.26] |
| GenderRecode |  |  |  |  | -0.02 | 0.01 | 0.00 | 0.00 |
|  |  |  |  |  | [-0.27, 0.23] | [-0.25, 0.27] | [-0.25, 0.25] | [-0.25, 0.26] |
| Bachelors |  |  |  |  | 0.26 * | 0.23 | 0.25 * | 0.25 |
|  |  |  |  |  | [0.00, 0.51] | [-0.03, 0.49] | [0.00, 0.51] | [-0.01, 0.50] |
| followers |  |  |  |  | 0.06 | 0.06 | 0.07 | 0.06 |
|  |  |  |  |  | [-0.10, 0.21] | [-0.10, 0.22] | [-0.09, 0.22] | [-0.09, 0.22] |
| friends |  |  |  |  | -0.07 | -0.08 | -0.08 | -0.07 |
|  |  |  |  |  | [-0.23, 0.09] | [-0.24, 0.08] | [-0.24, 0.08] | [-0.23, 0.09] |
| N | 460 | 460 | 460 | 460 | 231 | 231 | 231 | 231 |
| R2 | 0.01 | 0.00 | 0.02 | 0.03 | 0.15 | 0.12 | 0.15 | 0.15 |

Note: above are regression models examining how the number of Republican Accounts, UK Conservative Accounts, Hyperpartisan Accounts, and NewsGuard “Low Quality” accounts one follows predict vaccine confidence without (Models 1-4) and with (Models 5-8) control variables.

**Table S3.** Study 1 Supplementary Regression Models

|  | Model 1 | Model 2 | Model 3 | Model 4 | Model 5 | Model 6 | Model 7 | Model 8 |
| --- | --- | --- | --- | --- | --- | --- | --- | --- |
| (Intercept) | 0.00 | 0.00 | 0.00 | 0.00 | 0.01 | -0.00 | 0.00 | 0.00 |
|  | [-0.09, 0.09] | [-0.09, 0.09] | [-0.09, 0.09] | [-0.09, 0.09] | [-0.16, 0.18] | [-0.17, 0.17] | [-0.17, 0.18] | [-0.17, 0.17] |
| Republicans | -0.12 * |  |  |  | -0.14 * |  |  |  |
|  | [-0.21, -0.03] |  |  |  | [-0.26, -0.02] |  |  |  |
| Tory |  | 0.06 |  |  |  | 0.05 |  |  |
|  |  | [-0.04, 0.15] |  |  |  | [-0.07, 0.17] |  |  |
| HyperPartisan |  |  | -0.15 ** |  |  |  | -0.14 * |  |
|  |  |  | [-0.24, -0.06] |  |  |  | [-0.26, -0.02] |  |
| BadHandles |  |  |  | -0.19 *** |  |  |  | -0.15 * |
|  |  |  |  | [-0.28, -0.10] |  |  |  | [-0.27, -0.02] |
| Politics |  |  |  |  | -0.33 *** | -0.35 *** | -0.33 *** | -0.32 *** |
|  |  |  |  |  | [-0.46, -0.21] | [-0.48, -0.22] | [-0.45, -0.20] | [-0.45, -0.20] |
| GenderRecode |  |  |  |  | -0.02 | 0.00 | -0.01 | -0.00 |
|  |  |  |  |  | [-0.26, 0.22] | [-0.25, 0.25] | [-0.25, 0.23] | [-0.25, 0.24] |
| Age |  |  |  |  | 0.09 | 0.07 | 0.09 | 0.10 |
|  |  |  |  |  | [-0.03, 0.22] | [-0.05, 0.19] | [-0.03, 0.21] | [-0.02, 0.22] |
| N | 463 | 463 | 463 | 463 | 245 | 245 | 245 | 245 |
| R2 | 0.01 | 0.00 | 0.02 | 0.03 | 0.13 | 0.11 | 0.13 | 0.13 |

Note: above are regression models examining how the number of Republican Accounts, UK Conservative Accounts, Hyperpartisan Accounts, and NewsGuard “Low Quality” accounts one follows predict vaccine confidence without (Models 1-4) and with (Models 5-8) a different set of control variables used in a prior draft of this manuscript.

**Table S4.** Study 1 Additional Supplementary Regression Models

|  | Model 1 | Model 2 | Model 3 | Model 4 |
| --- | --- | --- | --- | --- |
| (Intercept) | 0.00 | 0.00 | 0.00 | 0.00 |
|  | [-0.09, 0.09] | [-0.09, 0.09] | [-0.09, 0.09] | [-0.09, 0.09] |
| UK Conservatives (Tories) Followed - UK Liberals (Labor Party) Followed | 0.02 |  |  |  |
|  | [-0.08, 0.11] |  |  |  |
| US Conservatives (Republicans) Followed - US Liberals (Democrats) Followed |  | -0.10 * |  |  |
|  |  | [-0.19, -0.01] |  |  |
| # of US Democrats Followed |  |  | 0.05 |  |
|  |  |  | [-0.04, 0.14] |  |
| # of UK Labor Party Members Followed |  |  |  | 0.06 |
|  |  |  |  | [-0.03, 0.16] |
| N | 460 | 460 | 460 | 460 |
| R2 | 0.00 | 0.01 | 0.00 | 0.00 |

Supplementary analysis showing how 1) the number of UK conservatives minus the number of UK liberals one follows predicts vaccine confidence, 2) how the number US conservatives minus the number of US liberals one follows predicts vaccine confidence, 3) how the number of Democrats one follows predicts vaccine confidence, and 4) how the number of labor party members one follows predicts vaccine confidence.

**Table S5.** Network Statistics

|  | **US Twitter Network** | | | **UK Twitter Network** | |
| --- | --- | --- | --- | --- | --- |
| **Complete Network Statistics** | | | | | |
| Average Path length | 3.398 | | | 3.285 | |
| Number of influencers followed by at least 3 participants | 614 | | | 2409 | |
| Density | 0.00496 | | | 0.00173 | |
| **Commonality Network Statistics** | | | | | |
| Average Path Length | 1.78 | | | 1.48 | |
| Density | 0.149 | | | 0.264 | |
| Modularity between political attitude communities | 0.0745 | | | 0.142 | |
| Modularity between vaccine attitude communities | 0.0067 | | | 0.003 | |
| Assortativity based on political conservatism | 0.167 | | | 0.032 | |
| Assortativity based on vaccine confidence | 0.049 | | | -0.013 | |
| **Community Level Statistics** | | | | | |
| Structural Communities | Community A | Community B | | Community A | Community B |
| Number of nodes (participants and “influencers”) | 583 | | 140 | 1728 | 799 |
| Number of participants | 95 | | 14 | 107 | 11 |
| Nominal assortativity between communities | 0.79 | | | 0.77 | |
| Average political conservatism among participants | 3.43 | | 6.07 | 4.41 | 5.09 |
|  | [3.00, 3.86] | | [5.80, 6.35] | [4.10, 4.72] | [4.33, 5.85] |
| Community difference in political conservatism | -2.64*** | | | -0.68 NS | |
|  | [-3.14, -2.14] | | | [-1.49, 0.13] | |
| Average vaccine confidence | 5.98 | | 4.46 | 6.06 | 6.50 |
|  | [5.72, 6.24] | | [3.45, 5.47] | [5.87, 6.25] | [6.24, 6.76] |
| Community difference in vaccine confidence | 1.51** | |  | -0.44** | |
|  | [0.48, 2.55] | |  | [-0.75, -0.13] | |
| **Node Level Statistics** | | | | | |
| Correlation between centrality within structural community and political conservatism | -0.23* | | 0.16 NS | -0.02 NS | 0.18 NS |
|  | [-0.40, -0.04] | | [-0.03, 0.33] | [-0.20, 0.16] | [-0.001, 0.35] |
| Correlation between centrality within structural community and vaccine confidence | 0.08 NS | | -0.22* | 0.07 NS | 0.15 NS |
|  | [-0.11, 0.27] | | [-0.39, -0.03] | [-0.12, 0.24] | [-0.04, 0.32] |
| Correlation between pairwise structural distance and pairwise difference in political conservatism | 0.08*** | | | 0.02 NS | |
|  | [0.06, 0.11] | | | [-0.002, 0.05] | |
| Correlation between pairwise structural distance and pairwise difference in vaccine confidence | 0.05*** | | | 0.04 ** | |
|  | [0.02, 0.07] | | | [0.01, 0.06] | |

*Note:* Network descriptive and inferential statistics in the US and the UK at the network level about the complete and the commonality networks, community level, and pair level, are shown above. “Complete Networks” refers to networks that contain both the participants and the influencers where links represent following relationships, “Commonality Networks” refers to networks that only contain the participants where links represent the number of accounts the pair of participants follow in common (See Supplementary Appendix Section S2). Community level refers to the statistics within each community in the US and the UK (Community A and Community B), and pair-level refers to the statistics about each pair of nodes in the US and UK networks. Assortativity based on an attitude measures the likelihood that connected nodes have a similar attitude; nominal assortativity measures the likelihood that connected nodes belong to the same cluster. 95% confidence intervals are shown in square brackets where applicable. NS denotes not significant, * denotes *p* < 0.05, ** denotes *p* < 0.01, *** denotes *p* < 0.001.

**Section S2.** Supplementary Network Analysis.

**Description of Network Statistics.** The average pathlength statistic is calculated by averaging the lengths of the shortest path between every pair of nodes in a network. A lower average pathlength reflects greater connectivity within a network. Here, a “path” refers to the number of edges connecting one node to another and does not refer to the absolute distance between two nodes on a graph visualization. Modularity is a measure for graph partitioning quality – in other words, the strength of division of a network into different sections, or modules. We assigned community memberships to nodes based on their self-reported political or vaccine attitudes (or for influencers, the average political or vaccine attitudes of the participants following them), and used the modularity coefficient to measure the extent to which political or vaccine opinion differences structurally partition the networks. The assortativity coefficient measures the likelihood that connected nodes have a similar attribute (i.e., political conservatism and vaccine confidence). We were not able to perform statistical tests for the differences between these descriptive statistics between the US and the UK. We also calculated the nominal assortativity between the communities in each country, or the likelihood that connected nodes belong to the same structural community.

**Commonality Networks.** Since we do not have information on influencers’ political attitudes or vaccine opinions, all the modularity and assortativity measures mentioned above are calculated from “Commonality Networks” that we constructed. Here, Commonality Networks are graphs where each node represents a participant, and each edge connecting two nodes represents that the two participants follow common influencers. The more influencers the two participants follow in common, the greater the strengths assigned to the edge. Edges are undirected since they represent mutual commonality. Thus, the Commonality Networks capture participants following similar accounts rather than direct follower connections. Since the graphs only contain participants whose political and vaccine attitudes are known from the surveys, we can compute the modularity and assortativity of the US and UK networks. ***Table S4*** reports the modularity and assortativity of the political and vaccine communities in these networks, along with other network statistics of densities and average path-lengths. The commonality networks are plotted visually in ***Figure S1***.


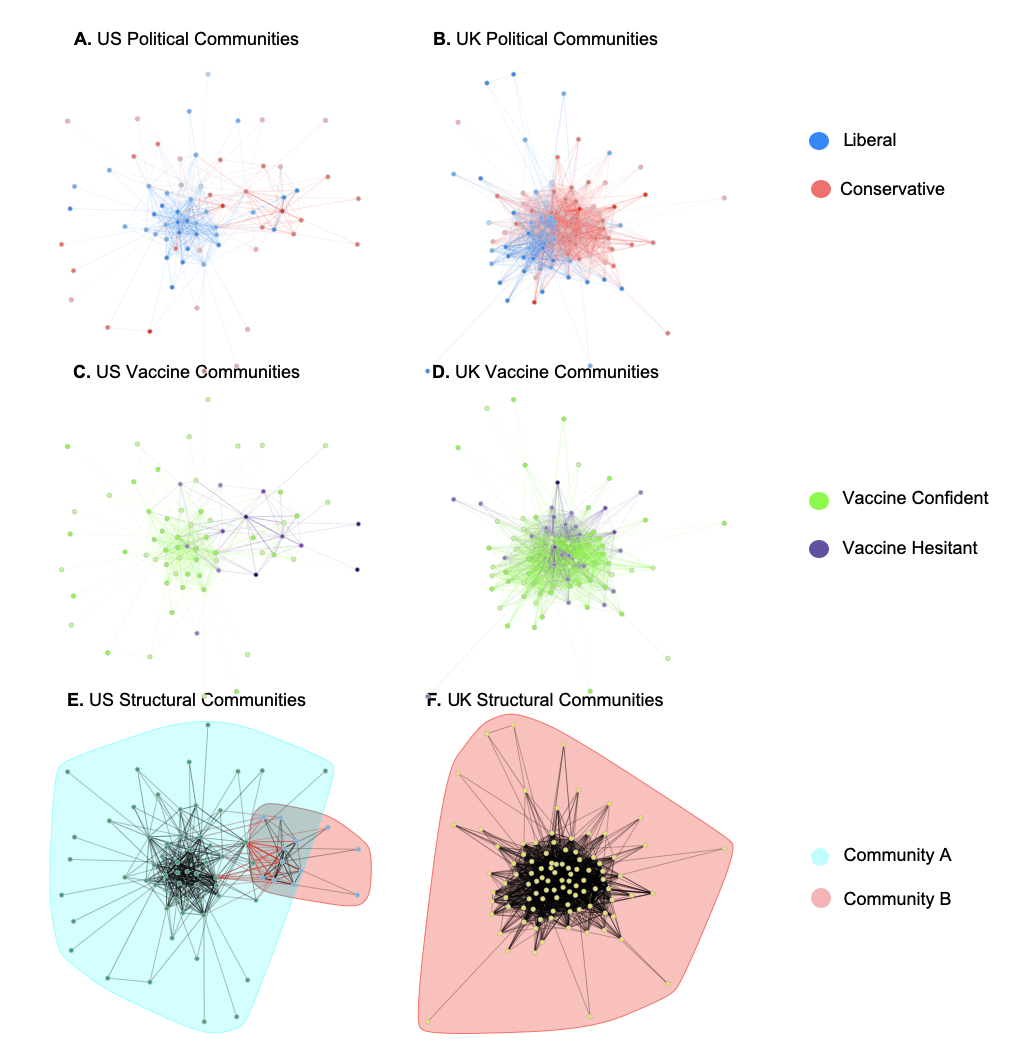


**Figure S1.** Commonality networks of the US and UK participants. Each node represents a participant, each edge between two nodes represents the pair of participants following at least 4 accounts in common, with the thickness (tie-strength) of the edges representing the number of accounts the pair follow in common. Tie-strengths are square-root-transformed to make the graphs more visually accessible, as some pairs of participants (particularly in the UK graphs) can follow many accounts in common. Nodes in Panel A and B are colored according to their self-reported political ideologies; nodes in Panel C and D are colored according to their self-reported vaccine attitudes. Panel E and F presents the results of a label-propagation graph partitioning analysis without prior assumption of the number of communities. The graph-partitioning algorithm identified two communities in the US Commonality Network, but only one in the UK.

**Different Network Sizes.** A visual inspection of the network visualizations in our main text suggests that the UK network is much larger than the US network. Density statistics of the US and UK complete networks show that both networks have very low density (US: 0.00496, UK: 0.00173). This is because the density statistics measure how connected the nodes are in a network, while in these networks no two participants share a link with each other, despite having followed many similar accounts. In the Commonality Networks, however, participants are directed connected based on their following commonality, and the density statistics were therefore meaningful in capturing the network characteristics, showing that the UK (0.264) is more densely connected than the US (0.149).

This visual disparity between the US and UK complete networks is because the UK has many more influencers than the US. With our exclusion criteria of eliminating influencers with less than 3 participants, the US has 614 influencers, while the UK has 2,409 influencers. We further found that while 90% of the “influencers” in the UK only have one follower among our participants, 95% of the US “influencers” only have one among our participants. This means that even by only filtering out the influencers followed by less than 2 participants, we lose more influencers in the US than in the UK. This difference in the effect of filtering, in addition to the US raw dataset already containing fewer influencers (despite more participants; see **Table S5** for details) than the UK, explains the apparent difference in the size of the US and the UK networks.

**Table S6.** Network size statistics under different influencer inclusion criteria

|  | **US** | | | **UK** | | |
| --- | --- | --- | --- | --- | --- | --- |
| Number of | Influencer | Participant | Edge | Influencer | Participant | Edge |
| N ≥ 5 | 150 | 99 | 1067 | 709 | 112 | 5417 |
| N ≥ 4 | 279 | 105 | 1583 | 1247 | 116 | 7569 |
| N ≥3 | 614 | 109 | 2588 | 2409 | 118 | 11055 |
| N ≥ 2 | 2414 | 117 | 6188 | 6465 | 120 | 19167 |
| N ≥ 1 | 46502 | 124 | 50276 | 64458 | 123 | 77160 |

*Node:* “N ≥x” denotes influencer inclusion criteria where influencers followed by less than x participants are excluded. “N 1” is the same as not filtering; “N 3” is the inclusion criteria chosen in the main analysis.

**Robustness Check of Influencer Inclusion Criteria in Network Analysis.** In the main analysis, we set an inclusion criterion that the nodes in the network graphs only include 1) influencers that are followed by at least 3 participants, and 2) participants following at least 1 influencer. To ensure that the inclusion criteria did not significantly alter the network structures, we conducted the following additional robustness check on how different inclusion criteria for influencers may impact the network topology.

First, we must acknowledge that, in the strictest definition, network topology will be changed when removing any node or edge from the network (1). Details of the network topology that are irrelevant to analysis, however, can be safely reduced without impacting the network topologies of interest, in our case, polarized communities. In fact, many graph partitioning algorithms that identify topological structures (or structural communities) in networks, such as the Girvan-Newman method (2), function by selectively removing nodes and edges to reduce topological details of the network and highlight structural communities. Our inclusion criteria for influencers aim to reduce structural details irrelevant to community detection while retaining as much relevant information as possible.

We can compare assortativity coefficients as a descriptive measure of structural separation of opinions (i.e., polarization) at different conditions, as shown in ***Table S7*.**

**Table S7.** Assortativity coefficients under different influencer inclusion criteria

|  | **Assortativity coefficient for political conservatism** | | **Assortativity coefficient for vaccine confidence** | |
| --- | --- | --- | --- | --- |
| Country | US | UK | US | UK |
| Including N $\geq$ 5 | 0.704 | 0.448 | 0.456 | 0.254 |
| Including N $\geq$ 4 | 0.716 | 0.506 | 0.495 | 0.315 |
| Including N $\geq$ 3 | 0.726 | 0.566 | 0.549 | 0.391 |
| Including N $\geq$ 2 | 0.773 | 0.670 | 0.690 | 0.531 |
| Including N $\geq$ 1 | 0.978 | 0.945 | 0.967 | 0.916 |

*Note:* “Including N $\geq x$” denotes inclusion criteria for influencers, where we filter out influencers that are followed by less than x participants. “Including N $\geq$ 1” is the same as not filtering, as all influencers are followed by at least one participant. “Including N $\geq$ 3” is our chosen inclusion criteria in the main analysis.

As shown in ***Table S8***, as inclusion criteria decrease, assortativity scores increase for both political conservatism and vaccine confidence in both countries. This is due to a lower inclusion criterion including more influencers that we assign attributes to based on their follower’s attributes, which inflates the likelihood that connected nodes will have similar attributes (the definition of assortativity). When not filtering, the assortativity coefficient approaches 1, because the majority of edges in the graph are participants following a lone “influencer” that is not followed by anyone else, meaning that most of the connected nodes will have similar attributes. Because we do not know the influencers’ attributes, we cannot perform network homophily or polarization analysis on a dataset where more than 90% of the attributes are inferred. However, it is worth noting that even though the UK has far more edges than the US under all inclusion criteria (**Table S5**), making it more likely to have connected nodes that have similar attributes, it appears to have lower assortativity than the US in all conditions. This shows that while changing inclusion criteria does change certain measures of network topology such as assortativity, the comparison between network topologies under the same criteria still reveals the same patterns of greater structural separation in the US.

**Table S8.** Network size statistics under different influencer inclusion criteria

|  | **US** | | | **UK** | | |
| --- | --- | --- | --- | --- | --- | --- |
| Number of | Influencer | Participant | Edge | Influencer | Participant | Edge |
| N >= 5 | 150 | 99 | 1067 | 709 | 112 | 5417 |
| N >= 4 | 279 | 105 | 1583 | 1247 | 116 | 7569 |
| N >= 3 | 614 | 109 | 2588 | 2409 | 118 | 11055 |
| N >= 2 | 2414 | 117 | 6188 | 6465 | 120 | 19167 |
| N >= 1 | 46502 | 124 | 50276 | 64458 | 123 | 77160 |

*Node:* “N >= x” denotes influencer inclusion criteria where influencers followed by less than x participants are excluded. “N >= 1” is the same as not filtering; “N >= 3” is the inclusion criteria chosen in the main analysis.

While certain topological details are lost during filtering of influencers, the structural communities remain consistent across different inclusion criteria. **Figure S2** demonstrates how different inclusion criteria impact the outcomes of community detection analysis in the networks, where stricter inclusion criteria reduce the size of networks visibly for both the US and the UK. Stricter criteria also disintegrate the larger cluster in the US (“Cluster A” in the main text) but not in the UK. However, we can see that the overall structural communities remain consistent under the same algorithmic graph partitioning, where only a few nodes in the US are partitioned into their own structural community. Therefore, structural communities remain relatively consistent under different inclusion criteria.

**Figure S2.** Changes of network visualizations under different influencer inclusion criteria. “Including N $\geq x$” denotes the influencer inclusion criteria, where influencers followed by less than $x$ participants are filtered out. “Including N $\geq$ 3” is the inclusion criteria used in the main analysis. Consistent with the main analysis, network graphs in this figure are created using the large-graph-layout algorithm in the igraph package in R; different graphs have slightly different layout orientations because they are created from different datasets under different inclusion criteria. Consistent with the main analysis, structural communities are identified using the same label-propagation graph partitioning algorithm. Lower inclusion criteria of $x$ = 1 or 2 are not visualized due to limited computational power.

**Additional Structural Polarization Analysis.** We also examined whether structural polarization in the network was related to participants’ belief polarization about politics and the vaccine. Specifically, we examined whether the structural distances between pairs of participants (i.e., the length of the shortest path between two nodes) predicts the attitudinal distances between them (i.e., the difference between two nodes’ levels of political conservatism and vaccine confidence). Since network dyadic statistics are not independent, we performed regressions between the adjacency matrices of the networks and the attitude-distance matrices and performed permutation tests (MRQAP, Dekker, 2007) on the results, instead of using traditional parametric tests on linear correlations. In the US, we found that pairwise structural distances were significantly associated with pairwise political attitude distances (ß = -0.192, *p* = .025) but not significantly associated with pairwise vaccine confidence distances (ß = -0.080, *p* = .177). In the UK, however, pairwise structural distances were not associated with pairwise political attitude distances (ß = 0.019, *p* = .398), but were significantly associated with pairwise vaccine confidence distances (ß = -0.113, *p* = .028). In other words, pairs of nodes that were structurally apart in the US network tend to also be apart in terms of politics but not vaccine confidence, while pairs of nodes that were structurally apart in the UK network tend to be apart in terms of vaccine confidence but not politics. Overall, these mixed results suggest that the US Twitter network is structurally separated along political lines but not vaccine attitude lines, and the UK Twitter network is structurally separated along vaccine attitude lines but not political lines.

**Different Centrality Statistics.** In ***Tables S9-S11***, we report results with different centrality measures as robustness checks (closeness centrality, eigenvector centrality, and betweenness centrality), and largely find the same results.

**Table S9.** Closeness Centrality

| Country | Community | Attitude | Correlation | t | p |
| --- | --- | --- | --- | --- | --- |
| US | A | Pol | r = -0.16 | t(107) = -1.65 | p = 0.101 |
| US | A | Vax | r = 0.18 | t(107) = 1.90 | p = 0.060 |
| US | B | Pol | r = 0.34 | t(107) = 3.70 | p < .001 |
| US | B | Vax | r = -0.25 | t(107) = -2.68 | p = 0.009 |
| UK | A | Pol | r = -0.04 | t(116) = -0.48 | p = 0.633 |
| UK | A | Pol | r = -0.02 | t(116) = -0.22 | p = 0.828 |
| UK | B | Vax | r = 0.10 | t(116) = 1.06 | p = 0.292 |
| UK | B | Pol | r = 0.05 | t(116) = 0.49 | p = 0.622 |

**Table S10.** Eigenvector Centrality

| Country | Community | Attitude | Correlation | t | p |
| --- | --- | --- | --- | --- | --- |
| US | A | Pol | r = -0.37 | t(107) = -4.15 | p < .001 |
| US | A | Vax | r = 0.20 | t(107) = 2.08 | p = 0.040 |
| US | B | Pol | r = 0.30 | t(107) = 3.27 | p = 0.001 |
| US | B | Vax | r = -0.24 | t(107) = -2.60 | p = 0.011 |
| UK | A | Pol | r = -0.03 | t(116) = -0.29 | p = 0.774 |
| UK | A | Pol | r = 0.06 | t(116) = 0.64 | p = 0.525 |
| UK | B | Vax | r = 0.18 | t(116) = 1.96 | p = 0.052 |
| UK | B | Pol | r = 0.13 | t(116) = 1.43 | p = 0.154 |

**Table S11.** Betweenness Centrality

| Country | Community | Attitude | Correlation | t | p |
| --- | --- | --- | --- | --- | --- |
| US | A | Pol | r = -0.23 | t(107) = -2.49 | p = 0.014 |
| US | A | Vax | r = 0.13 | t(107) = 1.31 | p = 0.193 |
| US | B | Pol | r = 0.24 | t(107) = 2.60 | p = 0.011 |
| US | B | Vax | r = -0.28 | t(107) = -3.04 | p = 0.003 |
| UK | A | Pol | r = 0.02 | t(116) = 0.20 | p = 0.842 |
| UK | A | Pol | r = 0.05 | t(116) = 0.53 | p = 0.594 |
| UK | B | Vax | r = 0.15 | t(116) = 1.65 | p = 0.101 |
| UK | B | Pol | r = 0.12 | t(116) = 1.33 | p = 0.186 |

**Potential Future Directions for Network Analysis.** As mentioned in the main discussion, our analysis is limited by the correlational nature of our methods and the observational nature of our datasets. Future work can attempt more advanced simulation methods to analyze echo chambers. One major limitation of our analysis is its inability to infer causality: while we showed that there is an association between attribute differences and structural separation in online social network, we were not able to make any claims about whether similar individuals self-selected to become structurally closer in the network, or individuals that are structurally closer in the network socially influenced each other to become more similar. Apart from using longitudinal data collected from randomized control trials to disentangle the causality, network science has produced numerous simulation methods through which one can potentially perform causal inferences on observational data (3, 4). For example, the exponential random graph model simulates potential network structures based on a set of given configurations (5), thereby allowing a statistical test to be performed on whether one node attribute (e.g., political conservatism) contributed significantly to the simulated formation of the observed network structure compared to a random graph, or compared to the contribution of another node attribute (e.g., vaccine confidence). Alternatively, the stochastic actor-oriented model (6) can simulate the real-time evolution of a network by simulating the formations of edges based on hypothesized node-similarities as new nodes enter the network, thereby allowing researchers to potentially compare simulated network evolution when a new attribute is added (e.g., vaccine opinions) with real-world time-stamped network observations.

**Table S12.** Influencers associated with low and high vaccine confidence in the US and the UK (using a threshold of influencers followed by 5+ participants).

| **Twitter Handle** | **Vaccine Confidence** | **% Getting Vaccinated** | **Twitter Handle** | **Vaccine Confidence** | **% Getting Vaccinated** |
| --- | --- | --- | --- | --- | --- |
| **Influencers Associated with Low Vaccine Confidence** | | | | | |
| PrisonPlanet | 1.83 (1.13) | 0.00 (0.00) | britishlibrary | 6.83 (0.26) | 100.00 (0.00) |
| Timcast | 2.43 (1.99) | 28.57 (48.80) | Metro_Ents | 6.80 (0.27) | 100.00 (0.00) |
| KatTimpf | 2.50 (1.90) | 16.67 (40.82) | ThePoke | 6.80 (0.27) | 100.00 (0.00) |
| laurenboebert | 2.58 (1.77) | 16.67 (40.82) | mackies_crisps | 6.80 (0.27) | 100.00 (0.00) |
| RudyGiuliani | 2.70 (1.04) | 0.00 (0.00) | fact_covid | 6.70 (0.45) | 100.00 (0.00) |
| EricTrump | 2.78 (1.64) | 11.11 (33.33) | OrendaBooks | 6.70 (0.27) | 100.00 (0.00) |
| parscale | 2.80 (1.89) | 20.00 (44.72) | educationgovuk | 6.70 (0.67) | 100.00 (0.00) |
| cvpayne | 2.83 (1.86) | 16.67 (40.82) | Tryanuary | 6.70 (0.27) | 100.00 (0.00) |
| RepMattGaetz | 2.86 (1.57) | 14.29 (37.80) | FitbitUK | 6.67 (0.41) | 100.00 (0.00) |
| RubinReport | 2.90 (2.07) | 20.00 (44.72) | JimHarris | 6.60 (0.55) | 100.00 (0.00) |
| ChanelRion | 2.90 (2.07) | 20.00 (44.72) | SouthernRailUK | 6.60 (0.65) | 100.00 (0.00) |
| TulsiGabbard | 2.90 (2.46) | 20.00 (44.72) | HeathrowAirport | 6.60 (0.42) | 100.00 (0.00) |
| MariaBartiromo | 2.92 (2.04) | 16.67 (40.82) | MensHealthUK | 6.60 (0.42) | 100.00 (0.00) |
| catturd2 | 2.93 (1.72) | 14.29 (37.80) | toyotires_uk | 6.60 (0.22) | 100.00 (0.00) |
| kimguilfoyle | 3.10 (1.64) | 20.00 (44.72) | DesignMuseum | 6.60 (0.89) | 100.00 (0.00) |
| **Influencers Associated with High Vaccine Confidence** | | | | | |
| CoryBooker | 6.90 (0.22) | 100.00 (0.00) | TomCruise | 3.40 (1.34) | 60.00 (54.77) |
| DrBiden | 6.79 (0.39) | 100.00 (0.00) | CapitalOfficial | 3.50 (1.26) | 16.67 (40.82) |
| ezraklein | 6.75 (0.42) | 100.00 (0.00) | JLSOfficial | 3.80 (1.15) | 60.00 (54.77) |
| AnnaKendrick47 | 6.71 (0.39) | 100.00 (0.00) | leonalewis | 3.80 (1.79) | 40.00 (54.77) |
| NotAltWorld | 6.70 (0.45) | 100.00 (0.00) | nicolerichie | 3.80 (1.79) | 60.00 (54.77) |
| Nate_Cohn | 6.70 (0.45) | 100.00 (0.00) | BrunoMars | 3.83 (1.44) | 55.56 (52.70) |
| ChrisEvans | 6.70 (0.45) | 100.00 (0.00) | PerezHilton | 3.83 (1.60) | 33.33 (51.64) |
| neilhimself | 6.67 (0.41) | 83.33 (40.82) | MrsSOsbourne | 3.83 (1.60) | 50.00 (54.77) |
| politico | 6.67 (0.41) | 100.00 (0.00) | BiffyClyro | 3.90 (1.14) | 60.00 (54.77) |
| VP | 6.62 (0.48) | 100.00 (0.00) | JamesGShore | 3.90 (1.29) | 40.00 (54.77) |
| NPR | 6.60 (0.42) | 100.00 (0.00) | HollyGShore | 3.90 (1.29) | 40.00 (54.77) |
| TheEconomist | 6.60 (0.55) | 100.00 (0.00) | Usher | 3.90 (1.43) | 40.00 (54.77) |
| LilNasX | 6.60 (0.55) | 80.00 (44.72) | mishacollins | 3.90 (1.85) | 60.00 (54.77) |
| Lin_Manuel | 6.60 (0.42) | 100.00 (0.00) | ddlovato | 3.94 (1.12) | 37.50 (51.75) |
| FLOTUS44 | 6.58 (0.49) | 100.00 (0.00) | freemoneylotto | 4.00 (1.17) | 0.00 (0.00) |

Note: In the above table, we replicated the analysis presented in Table 1 of the paper, showing the average vaccine confidence of influencers associated with low and high vaccine hesitancy, but this time we show influencers followed by at least 5 people.

**Table S13.** Influencers associated with low and high vaccine confidence in the US and the UK (using a threshold of influencers followed by 25+ participants).

| United States | | | United Kingdom | | |
| --- | --- | --- | --- | --- | --- |
| **Twitter Handle** | **Vaccine Confidence** | **% Getting Vaccinated** | **Twitter Handle** | **Vaccine Confidence** | **% Getting Vaccinated** |
| Influencers Associated with Low Vaccine Confidence | | | | | |
| elonmusk | 4.41 (2.07) | 55.17 (50.61) | RealDMitchell | 6.04 (0.89) | 92.86 (26.23) |
| BarackObama | 5.96 (1.37) | 85.71 (35.63) | daraobriain | 6.02 (0.88) | 100.00 (0.00) |
|  |  |  | SoVeryBritish | 5.91 (0.93) | 89.29 (31.50) |
|  |  |  | stephenfry | 5.90 (1.11) | 95.83 (20.19) |
|  |  |  | BBCNews | 5.82 (1.05) | 85.71 (35.63) |
|  |  |  | JoeBiden | 5.75 (1.30) | 88.46 (32.58) |
|  |  |  | jackwhitehall | 5.73 (0.98) | 91.89 (27.67) |
|  |  |  | MartinSLewis | 5.72 (1.08) | 87.50 (33.42) |
|  |  |  | GaryLineker | 5.71 (0.92) | 79.41 (41.04) |
|  |  |  | BBCBreaking | 5.69 (1.15) | 81.48 (39.21) |
|  |  |  | AldiUK | 5.67 (1.11) | 80.77 (40.19) |
|  |  |  | jeremycorbyn | 5.67 (1.21) | 83.33 (37.90) |
|  |  |  | rickygervais | 5.64 (1.18) | 84.62 (36.55) |
|  |  |  | piersmorgan | 5.59 (1.23) | 85.71 (35.63) |
|  |  |  | Twitter | 5.58 (1.39) | 90.00 (30.51) |
| Influencers Associated with High Vaccine Confidence | | | | | |
| BarackObama | 5.96 (1.37) | 85.71 (35.63) | hollywills | 5.22 (1.51) | 80.00 (40.51) |
| elonmusk | 4.41 (2.07) | 55.17 (50.61) | davidwalliams | 5.30 (1.25) | 74.07 (44.66) |
|  |  |  | Fearnecotton | 5.33 (1.33) | 88.46 (32.58) |
|  |  |  | elonmusk | 5.40 (1.48) | 85.29 (35.95) |
|  |  |  | antanddec | 5.41 (1.26) | 78.95 (41.32) |
|  |  |  | AmazonUK | 5.48 (1.36) | 75.00 (44.10) |
|  |  |  | jk_rowling | 5.48 (1.62) | 82.14 (39.00) |
|  |  |  | JKCorden | 5.48 (1.09) | 84.85 (36.41) |
|  |  |  | Lord_Sugar | 5.50 (1.33) | 85.71 (35.63) |
|  |  |  | jimmycarr | 5.50 (1.24) | 92.31 (27.17) |
|  |  |  | JeremyClarkson | 5.50 (0.93) | 90.32 (30.05) |
|  |  |  | BorisJohnson | 5.52 (1.37) | 85.71 (35.42) |
|  |  |  | BarackObama | 5.53 (1.36) | 82.61 (38.32) |
|  |  |  | VancityReynolds | 5.53 (1.49) | 76.67 (43.02) |
|  |  |  | SkyNews | 5.53 (1.31) | 83.33 (37.90) |

Note: In the above table, we replicated the analysis presented in Table 1 of the paper, showing the average vaccine confidence of influencers associated with low and high vaccine hesitancy, but this time we show influencers followed by at least 25 people. Note that in the US, only two influencers (Barack Obama and Elon Musk) are followed by more than 25 people.

**Table S14.** Study 2 Regression Models

|  | Hyperpartisan | Hyperpartisan 2 | NewsGuard Shares | NewsGuard Shares 2 | NewsGuard Favorites | NewsGuard Favorites 2 |
| --- | --- | --- | --- | --- | --- | --- |
| (Intercept) | 0.00 | 0.09 | 0.00 | -0.43 ** | 0.00 | -0.33 ** |
|  | [-0.05, 0.05] | [-0.11, 0.28] | [-0.06, 0.06] | [-0.69, -0.16] | [-0.06, 0.06] | [-0.58, -0.08] |
| vaccineLikely | -0.07 ** | -0.10 * | 0.23 *** | 0.19 *** | 0.23 *** | 0.11 * |
|  | [-0.12, -0.02] | [-0.18, -0.02] | [0.17, 0.29] | [0.09, 0.29] | [0.17, 0.28] | [0.02, 0.21] |
| Liberalism |  | 0.03 |  | 0.08 |  | 0.24 *** |
|  |  | [-0.06, 0.12] |  | [-0.03, 0.18] |  | [0.13, 0.35] |
| Polarization |  | 0.08 |  | -0.03 |  | -0.05 |
|  |  | [-0.00, 0.16] |  | [-0.13, 0.07] |  | [-0.15, 0.05] |
| Conspiracy |  | 0.01 |  | -0.05 |  | -0.12 ** |
|  |  | [-0.07, 0.09] |  | [-0.14, 0.04] |  | [-0.21, -0.03] |
| mentalHealth |  | -0.07 |  | 0.00 |  | -0.00 |
|  |  | [-0.17, 0.03] |  | [-0.12, 0.12] |  | [-0.12, 0.12] |
| lifeSatisfaction |  | -0.02 |  | 0.04 |  | -0.07 |
|  |  | [-0.11, 0.08] |  | [-0.08, 0.16] |  | [-0.18, 0.05] |
| Male |  | -0.03 |  | 0.03 |  | 0.06 |
|  |  | [-0.18, 0.12] |  | [-0.15, 0.21] |  | [-0.12, 0.24] |
| Age |  | 0.21 *** |  | 0.04 |  | 0.08 |
|  |  | [0.14, 0.29] |  | [-0.05, 0.13] |  | [-0.02, 0.17] |
| Bachelors |  | -0.09 |  | 0.48 *** |  | 0.35 ** |
|  |  | [-0.28, 0.10] |  | [0.22, 0.74] |  | [0.11, 0.59] |
| followers_count |  | -0.02 |  | 0.06 |  | 0.03 |
|  |  | [-0.10, 0.06] |  | [-0.05, 0.18] |  | [-0.08, 0.14] |
| friends_count |  | 0.11 ** |  | 0.05 |  | -0.05 |
|  |  | [0.03, 0.19] |  | [-0.06, 0.17] |  | [-0.16, 0.06] |
| N | 1600 | 734 | 1036 | 475 | 1064 | 480 |
| R2 | 0.00 | 0.08 | 0.05 | 0.13 | 0.05 | 0.16 |

Note: Study 2 regression models, showing (from left-right) how one’s likelihood of vaccine predicts the sharing of Hyperpartisan news (with and without controls), how one’s likelihood of getting the vaccine predicts the quality of news URLS shared as rated by NewsGuard (with and without controls), and how one’s likelihood of getting the vaccine predicts the quality of news URLs favorited as rated by NewsGuard (with and without controls).

**Table S15.** Study 2 Regression Models With Different Control Variables

|  | Hyperpartisan | Hyperpartisan 2 | NewsGuard Shares | NewsGuard Shares 2 | NewsGuard Favorites | NewsGuard Favorites 2 |
| --- | --- | --- | --- | --- | --- | --- |
| (Intercept) | 0.00 | 0.01 | 0.00 | -0.05 | 0.00 | -0.04 |
|  | [-0.05, 0.05] | [-0.07, 0.10] | [-0.06, 0.06] | [-0.16, 0.06] | [-0.06, 0.06] | [-0.15, 0.07] |
| vaccineLikely | -0.07 ** | -0.14 *** | 0.23 *** | 0.18 *** | 0.23 *** | 0.17 *** |
|  | [-0.12, -0.02] | [-0.20, -0.07] | [0.17, 0.29] | [0.10, 0.26] | [0.17, 0.28] | [0.09, 0.24] |
| Liberalism |  | 0.04 |  | 0.16 *** |  | 0.20 *** |
|  |  | [-0.02, 0.10] |  | [0.08, 0.24] |  | [0.12, 0.27] |
| Male |  | -0.02 |  | 0.09 |  | 0.06 |
|  |  | [-0.14, 0.09] |  | [-0.06, 0.24] |  | [-0.08, 0.21] |
| Age |  | 0.20 *** |  | 0.07 * |  | 0.07 |
|  |  | [0.14, 0.26] |  | [0.00, 0.15] |  | [-0.00, 0.14] |
| N | 1600 | 1093 | 1036 | 690 | 1064 | 705 |
| R2 | 0.00 | 0.05 | 0.05 | 0.08 | 0.05 | 0.10 |

Note: Study 2 regression models, showing (from left-right) how one’s likelihood of vaccine predicts the sharing of hyperpartisan news (with and without controls), how one’s likelihood of getting the vaccine predicts the quality of news URLS shared as rated by NewsGuard (with and without controls), and how one’s likelihood of getting the vaccine predicts the quality of news URLs favorited as rated by NewsGuard (with and without controls). This table shows different control variables that were used in an earlier draft of the manuscript.

**Table S16.** Specific URLs shared or favorited associated with low self-reported likelihood of receiving the vaccine (using threshold of 25+ shares or favorites)

| **News Website Shared** | **Likelihood of Getting Vaccine** | **News Website Favorited** | **Likelihood of Getting Vaccine** |
| --- | --- | --- | --- |
| espn.com | 88.00 (30.37) | foxnews.com | 88.21 (30.62) |
| dailymail.co.uk | 88.84 (27.76) | metro.co.uk | 88.94 (27.43) |
| metro.co.uk | 89.80 (25.55) | dailymail.co.uk | 89.81 (27.00) |
| mashable.com | 90.82 (23.72) | nypost.com | 90.09 (25.55) |
| boingboing.net | 90.87 (25.05) | variety.com | 90.91 (25.74) |
| msn.com | 92.20 (27.01) | chicago.suntimes.com | 91.85 (19.92) |
| nypost.com | 92.35 (23.98) | standard.co.uk | 91.93 (23.63) |
| on.wsj.com | 92.47 (22.64) | a.msn.com | 92.06 (24.02) |
| huffingtonpost.co.uk | 92.69 (20.90) | chicagotribune.com | 92.14 (23.61) |
| on.ft.com | 92.72 (22.45) | truthout.org | 92.19 (27.15) |
| salon.com | 93.27 (20.72) | thesun.co.uk | 92.31 (21.87) |
| foxnews.com | 93.41 (21.90) | deadline.com | 92.42 (24.17) |
| nationalreview.com | 93.57 (23.76) | mol.im | 92.50 (23.84) |
| ajc.com | 93.60 (22.89) | yahoo.com | 92.67 (23.34) |
| dailykos.com | 93.62 (22.43) | people.com | 92.67 (23.80) |

Note: In the above table, we replicate the analysis shown in Table 2, but show URLs that are shared or favorited by at least 25 participants.

**Table S17.** Specific URLs shared or favorited associated with low self-reported likelihood of receiving the vaccine (using threshold of 5+ shares or favorites)

| News Website Shared | Likelihood of Getting Vaccine | News Website Favorited | Likelihood of Getting Vaccine |
| --- | --- | --- | --- |
| redstate.com | 58.00 (53.10) | thegatewaypundit.com | 45.44 (45.42) |
| 6abc.com | 61.40 (52.91) | chroniclelive.co.uk | 54.60 (42.61) |
| breitbart.com | 65.78 (43.88) | cancer.gov | 60.00 (54.77) |
| thepostmillennial.com | 66.00 (34.89) | newsmax.com | 66.71 (46.03) |
| billboard.com | 69.08 (42.21) | billboard.com | 70.07 (41.76) |
| zerohedge.com | 72.62 (42.52) | deviantart.com | 72.30 (41.00) |
| dailycaller.com | 72.86 (46.45) | westernjournal.com | 72.40 (43.69) |
| dailywire.com | 72.86 (43.48) | zerohedge.com | 73.94 (39.67) |
| thewrap.com | 73.00 (43.53) | thepostmillennial.com | 75.23 (38.98) |
| fox32chicago.com | 73.40 (39.88) | khou.com | 77.00 (40.67) |
| foxbusiness.com | 73.50 (41.05) | realclearpolitics.com | 77.00 (40.67) |
| crooksandliars.com | 74.40 (35.17) | thewrap.com | 77.73 (38.93) |
| washingtonexaminer.com | 75.92 (35.16) | breitbart.com | 78.07 (37.21) |
| theepochtimes.com | 76.67 (40.82) | tvline.com | 78.33 (43.01) |
| treehugger.com | 76.67 (31.80) | fox26houston.com | 79.20 (44.31) |

Note: In the above table, we replicate the analysis shown in Table 2, but show URLs that are shared or favorited by at least 5 participants.

**
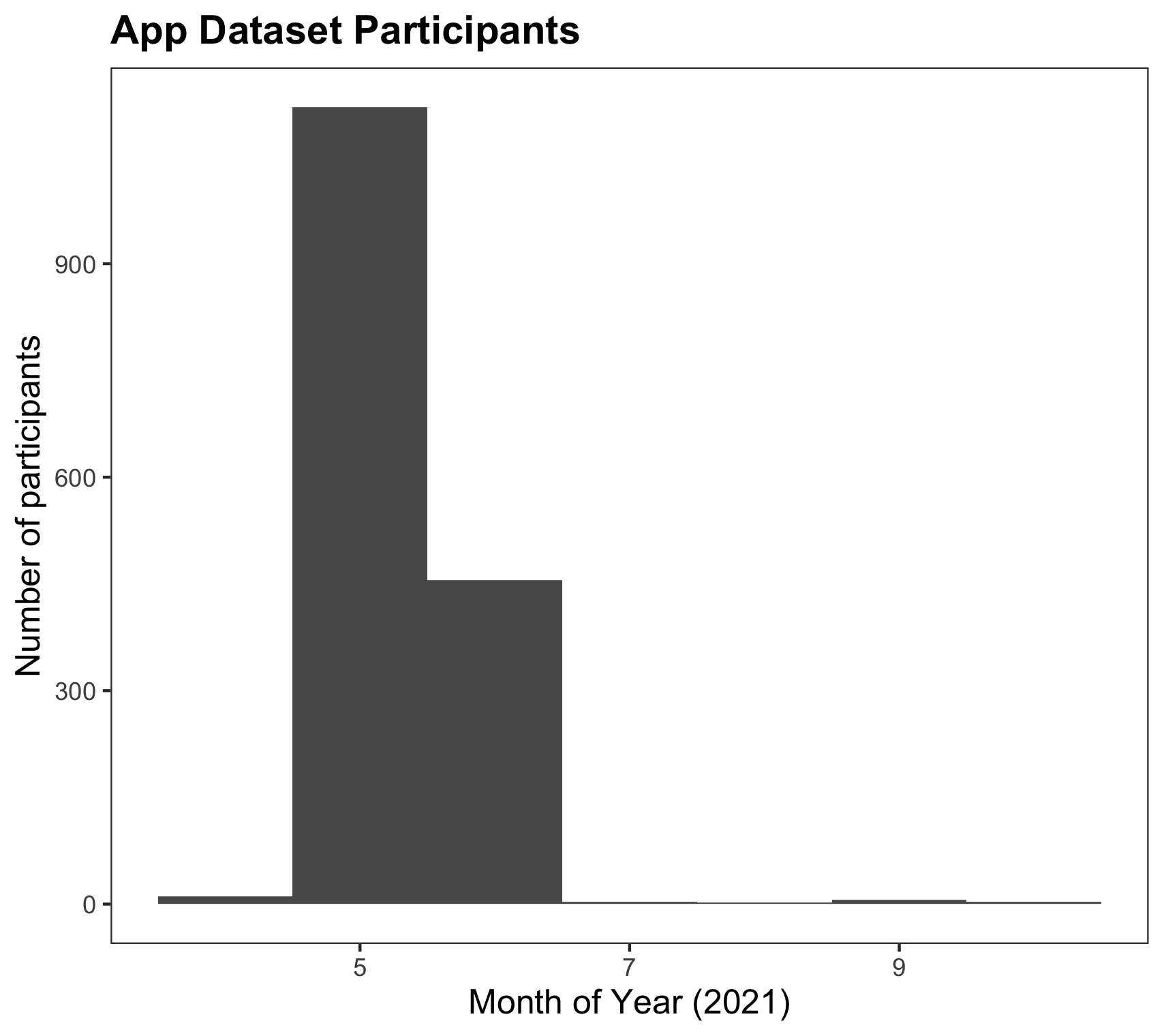
**

**Figure S3.** Histogram of when app participants were recruited. Most participants were recruited when the app was first sent out on Twitter in May, 2021. However, a second wave of participants also used the app when Study 1 participants were recommended to use it in June, 2021.

**Supplemental References**

1. D. Easley, J. Kleinberg, *Networks, crowds, and markets: Reasoning about a highly connected world* (Cambridge university press, 2010).

2. M. Girvan, M. E. Newman, Community structure in social and biological networks. *Proceedings of the national academy of sciences* **99**, 7821–7826 (2002).

3. M. McPherson, L. Smith-Lovin, J. M. Cook, Birds of a feather: Homophily in social networks. *Annual review of sociology* **27**, 415–444 (2001).

4. S. Aral, L. Muchnik, A. Sundararajan, Distinguishing influence-based contagion from homophily-driven diffusion in dynamic networks. *Proceedings of the National Academy of Sciences* **106**, 21544–21549 (2009).

5. G. Robins, P. Pattison, Y. Kalish, D. Lusher, An introduction to exponential random graph (p*) models for social networks. *Social networks* **29**, 173–191 (2007).

6. T. A. Snijders, Stochastic actor-oriented models for network dynamics. *Annual review of statistics and its application* **4**, 343–363 (2017).
